# H_2_S‐Releasing Aspirin Nanoparticles Alleviate Endometriosis and Associated Anxiety

**DOI:** 10.1002/advs.202520787

**Published:** 2026-02-08

**Authors:** Mengni Zhou, Renbin Dou, Rong Wu, Yunyu Xu, Ying Wang, Peng Wang, Jinfu Li, Pengcheng Lu, Yu Mao, Jieying Qian, Yunjiao Zhang, Jiqian Zhang, Shasha Zhu

**Affiliations:** ^1^ Department of Obstetrics and Gynecology Engineering Research Center of Biopreservation and Artificial Organs the First Affiliated Hospital of Anhui Medical University NHC Key Laboratory of Study on Abnormal Gametes and Reproductive Tract (Anhui Medical University) Ministry of Education Hefei Anhui China; ^2^ Lu'an People's Hospital of Anhui Province Lu'an Hospital of Anhui Medical University Lu'an Anhui China; ^3^ Department of Anesthesiology Key Laboratory of Anesthesiology and Perioperative Medicine of Anhui Higher Education Institutes the First Affiliated Hospital of Anhui Medical University Anhui Medical University Hefei Anhui China; ^4^ School of Medicine South China University of Technology Guangzhou Guangdong China; ^5^ National Engineering Research Center For Tissue Restoration and Reconstruction and Key Laboratory of Biomedical Engineering of Guangdong Province South China University of Technology Guangzhou Guangdong China

**Keywords:** anxiety‐like behavior, bovine serum albumin, endometriosis, gasotransmitter, hydrogen sulfide, nanoparticle

## Abstract

Despite ongoing challenges in developing effective non‐surgical and non‐hormonal treatments for endometriosis, the psychological manifestations of the disease—particularly anxiety—remain comparatively underexplored. In this study, a hydrogen sulfide (H_2_S)‐releasing aspirin derivative, ACS14, was encapsulated in albumin nanoparticles (ACS14@BSA) for targeted delivery. Upon intraperitoneal injection in a mouse model of endometriosis, ACS14@BSA was selectively transported to ectopic lesions via a neutrophil hitchhiking strategy. There, it inhibited ectopic cell proliferation by modulating the PI3K/Akt pathway and reduced inflammation by suppressing the NF‐κB pathway, exerting a comprehensive therapeutic effect on endometriosis. Simultaneously, the H_2_S released at lesion sites was conveyed through the bloodstream to the anterior cingulate cortex (ACC), a brain region critical for anxiety regulation, as demonstrated by in vivo fiber photometry recordings in mice. Importantly, in the ACC, H_2_S upregulated glutamate transporter 1 (GLT‐1), decreased extracellular glutamate levels, and dampened glutamatergic neuron hyperactivity, thereby alleviating endometriosis‐associated anxiety. This study presents a novel gasotransmitter‐releasing nanoplatform that offers a non‐invasive and non‐hormonal approach for the concurrent treatment of endometriosis and associated anxiety.

## Graphical abstract

Albumin nanoparticles loaded with an H_2_S‐releasing aspirin derivative (ACS14) hitchhiking neutrophils after intraperitoneal injection and preferentially accumulate in endometriosis lesions in mice. The treatment suppresses lesion growth by regulating PI3K/Akt and reduces inflammation by inhibiting NF‐κB. Meanwhile, H_2_S reaching the anterior cingulate cortex lowers glutamatergic neuron activity and alleviates endometriosis‐associated anxiety.

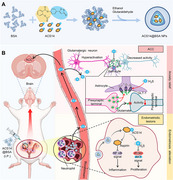

## 1 Introduction

Endometriosis is a common gynecological inflammatory disorder characterized by the ectopic presence of endometrial‐like tissue outside the uterus. Affecting approximately 10% of women of reproductive age worldwide, endometriosis is a leading cause of chronic pelvic pain and infertility [1, 2]. Current treatment strategies are largely limited to surgical excision and hormonal modulation. However, recurrence rates following surgery remain high—up to 21.5% within two years and 40%–50% within five years—while hormonal therapies are associated with adverse effects, including mood disturbances, pseudo‐menopausal symptoms, loss of bone density, and elevated risk of osteoporosis [3].To overcome these limitations, a number of nanotechnology‐based therapeutic approaches have been evaluated in preclinical models, including photothermal therapy, gene therapy, and targeted hormone delivery, all of which have demonstrated encouraging efficacy [4, 5]. Nonetheless, minimally invasive, non‐hormonal treatments with improved safety profiles remain underdeveloped. Among emerging strategies, albumin‐based nanocarriers have shown promise due to their ability to selectively accumulate at ectopic lesions in mouse models of endometriosis [6]. This targeted delivery is facilitated by mechanisms such as neutrophil hitchhiking and SPARC (secreted protein acidic and rich in cysteine)‐mediated uptake [6, 7]. Given the established clinical translation of albumin‐based nanomedicines (e.g., nab‐paclitaxel and nab‐sirolimus) in oncology and other therapeutic areas, albumin‐based delivery systems may provide a translatable platform for endometriosis therapy [8, 9]. However, investigations into albumin‐based strategies specifically for endometriosis remain limited.

In addition to physical symptoms, endometriosis is frequently accompanied by psychological comorbidities, most notably anxiety, which can substantially complicate disease management [10, 11]. While often underdiagnosed, psychiatric disorders affect a notable proportion of patients, with approximately 7% requiring hospitalization for severe symptoms [12]. Anxiety is the most common comorbidity and has been associated with poorer clinical outcomes, increased disease burden, and diminished quality of life [13]. Despite this high prevalence, there are currently no effective therapeutic approaches that simultaneously target both endometriosis and its associated anxiety. Management of anxiety in these patients typically involves pharmacotherapy, most commonly selective serotonin reuptake inhibitors (SSRIs), and, in some cases, benzodiazepines [14]. However, these agents primarily provide symptomatic relief, do not address the underlying pathology of endometriosis, and are associated with adverse effects, including sleep disruption and potential long‐term cognitive risks [15, 16]. Collectively, these limitations underscore the need for therapeutic strategies capable of safely and effectively addressing both the physical and psychological dimensions of endometriosis, thereby improving overall patient outcomes.

Gaseous signaling molecules (GSMs), including hydrogen sulfide (H_2_S), carbon monoxide (CO), and nitric oxide (NO), function as gasotransmitters involved in organ development, tissue homeostasis, and the modulation of disease processes [17, 18, 19]. Among them, H_2_S is capable of crossing the blood‐brain barrier and has demonstrated rapid anxiolytic and antidepressant effects in various disease models, largely attributed to its ability to mitigate oxidative stress [20, 21]. To overcome challenges related to the storage and controlled release of GSMs, a variety of H_2_S donor molecules have been developed. One such compound, the H_2_S‐releasing aspirin derivative ACS14 (2‐acetyloxybenzoic acid 4‐(3‐thioxo‐3H‐1,2‐dithiol‐5‐yl) phenyl ester), simultaneously delivers both H_2_S and aspirin [22]. ACS14 has shown efficacy in treating pulmonary arterial hypertension, modulating vascular remodeling, and exerting antithrombotic effects [23, 24]. Beyond its cardiovascular effects, H_2_S can inhibit cell proliferation and promote apoptosis by suppressing the PI3K/Akt signaling pathway [25, 26], while aspirin is a widely used anti‐inflammatory agent that exerts its effects through multiple mechanisms, including inhibition of the NF‐κB pathway [27, 28, 29]. Notably, vascular inflammation is an important component of endometriosis pathophysiology and a potentially actionable therapeutic dimension [30, 31]. Collectively, these combined properties suggest that ACS14 may serve as a promising dual‐function therapeutic for the treatment of endometriosis and its comorbid anxiety, a potential that merits further investigation.

**FIGURE 1:**
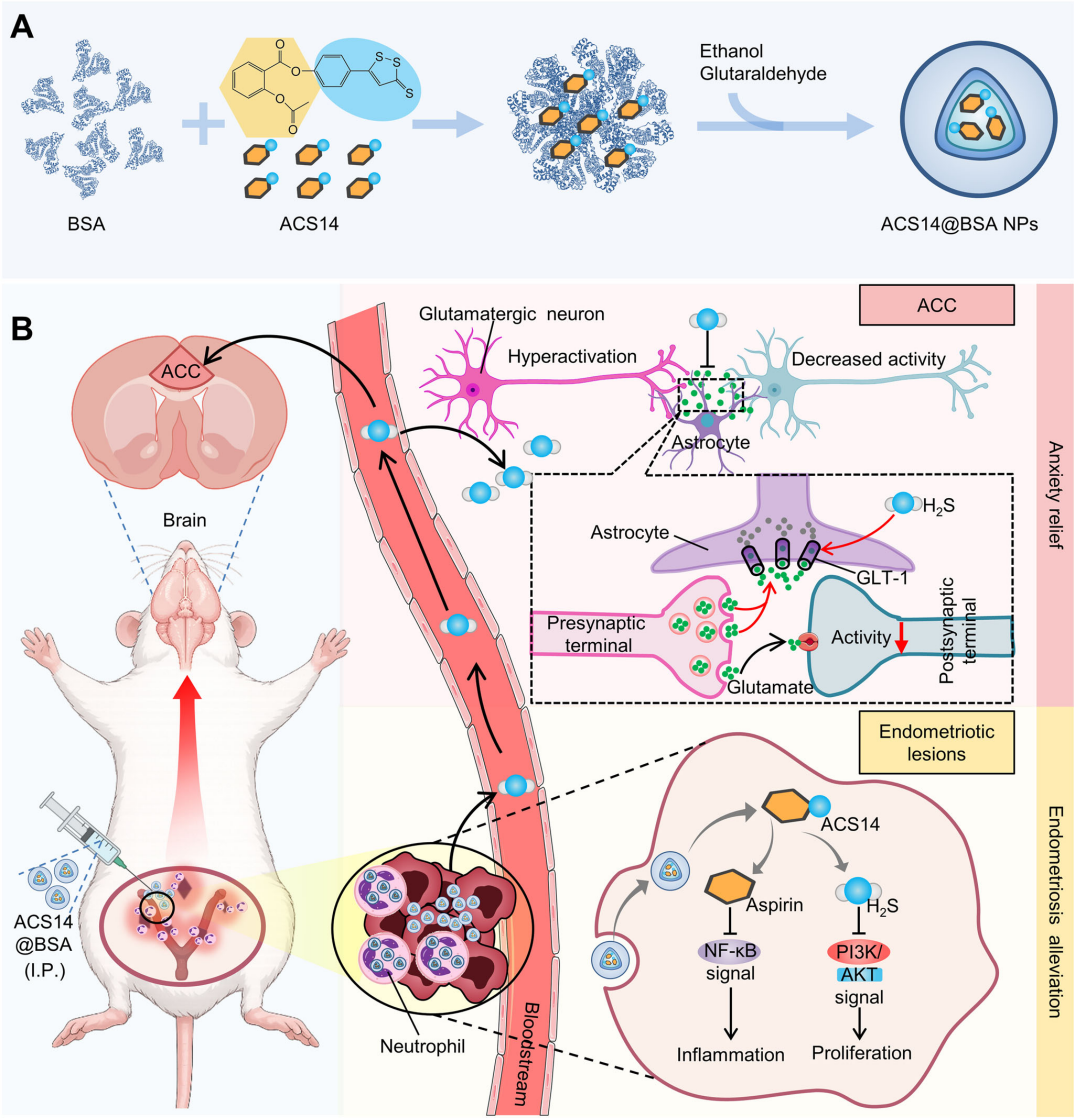
Schematic illustration of the mechanisms by which ACS14@BSA exerts anti‐endometriosis effects while concurrently alleviating anxiety‐like behaviors. Following intraperitoneal injection, ACS14@BSA was delivered to ectopic lesions via neutrophil hitchhiking, where it inhibited cell proliferation through PI3K/Akt modulation and reduced inflammation via NF‐κB suppression, exerting anti‐endometriotic effects. Meanwhile, H_2_S released at the lesion site entered the bloodstream and was transported to the anterior cingulate cortex, where it upregulated glutamate transporter 1 (GLT‐1), decreased extracellular glutamate, and suppressed glutamatergic neuron hyperactivity, thereby alleviating endometriosis‐associated anxiety.

Given that current H_2_S donors, when directly administered intraperitoneally in vivo [32, 33], suffer from suboptimal gas release profiles and associated side effects leading to poor efficacy [19, 34, 35], we loaded ACS14 into albumin nanoparticles (ACS14@BSA) to overcome these limitations. Following intraperitoneal injection in a mouse model of endometriosis, ACS14@BSA was specifically delivered to the ectopic lesions via a neutrophil hitchhiking approach [6]. Subsequently, ACS14@BSA inhibited ectopic cell proliferation by modulating the PI3K/Akt pathway and reduced inflammation through suppression of the NF‐κB pathway, thereby exerting a comprehensive inhibitory effect on endometriosis. Additionally, given that pain is a major driver of anxiety in endometriosis [11, 36] and that the anterior cingulate cortex (ACC) serves as a key hub for pain–affect integration [37, 38], with glutamatergic neurons playing a central role [38, 39, 40], we elucidated an ACC‐dependent neurobiological pathway in ACS14@BSA‐treated endometriosis mice. We found that the H_2_S released at the lesion site was transported through the bloodstream to the ACC, where it upregulated glutamate transporter 1 (GLT‐1), reduced extracellular glutamate levels, and attenuated the hyperactivity of glutamatergic neurons, thereby alleviating endometriosis‐associated anxiety (Figure 1). Our study presents a novel gasotransmitter‐mediated therapeutic strategy, providing a promising non‐invasive and non‐hormonal avenue for the concurrent treatment of endometriosis and associated anxiety.

## 2 Results and Discussion

### 2.1 Preparation and Characterization of ACS14@BSA Nanoparticles

**FIGURE 2:**
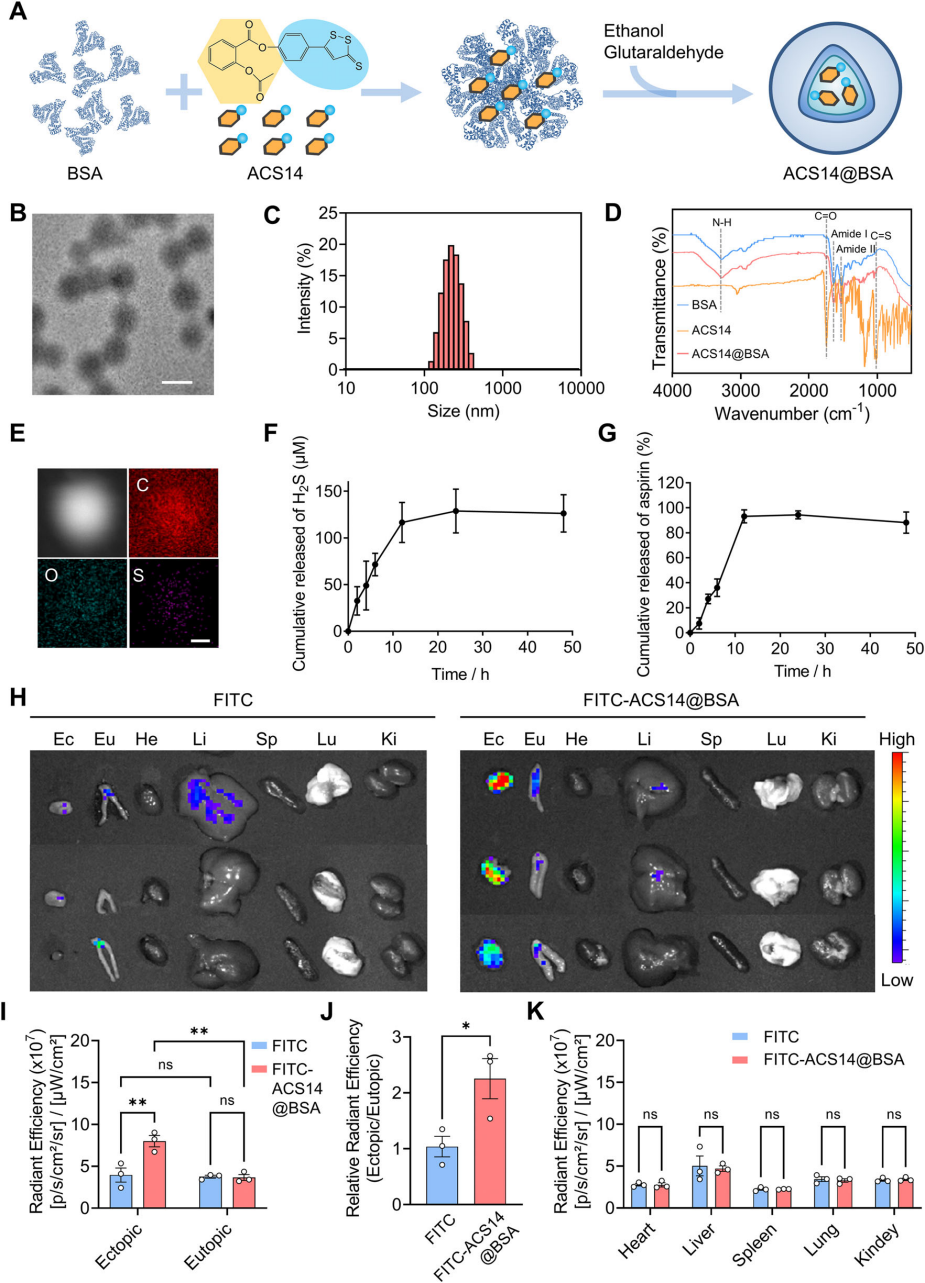
Characterization of ACS14@BSA. (A) Schematic illustration for the preparation of ACS14@BSA. (B) Representative TEM image of ACS14@BSA. Scale bar: 200 nm. (C) The size distribution of ACS14@BSA. (D) The FTIR transmittance spectra of ACS14@BSA nanoparticles, ACS14, and BSA nanoparticles. (E) EDX mapping image of ACS14@BSA nanoparticles shows that ACS14@BSA contains the elements C, O, and S. Scale bar: 50 nm. (F) In vitro cumulative release of H_2_S and (G) aspirin from ACS14@BSA over 48 h in PBS at pH 5.5 (*n* = 3). (H) Ex vivo fluorescence images of eutopic endometrium, ectopic lesions, and major organs collected 16 h after intraperitoneal injection of free FITC or FITC‐ACS14@BSA. (I) Quantification of average fluorescence intensities in the eutopic endometrium and ectopic lesions (*n* = 3). (J) Ratio of ectopic/eutopic fluorescence (*n* = 3). (K) Quantification of average fluorescence intensities in the heart (He), liver (Li), spleen (Sp), lung (Lu), and kidney (Ki) (*n* = 3). Data are shown as the mean ± SEM. ^*^ *p* < 0.05, ^**^ *p* < 0.01; ns, not significant.

To enable targeted delivery to endometriotic lesions with strong translational potential [6, 8], albumin‐based nanoparticles encapsulating ACS14 (ACS14@BSA) were prepared using a simple, low‐cost, and highly reproducible method (Figure 2A; Figure S1). Transmission electron microscopy (TEM) revealed that the nanoparticles generally exhibited a relatively uniform spherical morphology (Figure 2B). Dynamic light scattering (DLS) analysis showed an average hydrodynamic diameter of approximately 219.5 ± 4.2 nm (Figure 2C). Fourier transform infrared spectroscopy (FTIR) was employed to analyze the transmittance spectrum of ACS14@BSA nanoparticles, blank BSA nanoparticles, and ACS14 powder over the range of 500–4000 cm^−^^1^. As shown in Figure 2D, the FTIR transmittance spectrum of ACS14@BSA retains the characteristic BSA bands (3274 cm^−^^1^ for N─H stretching and the amide I/II bands at 1635 and 1517 cm^−^^1^) [41, 42, 43] and also exhibits ACS14‐specific peaks (1746 cm^−^^1^ for Ar─C═O and 1014 cm^−^^1^ for C═S) [23, 44], supporting the incorporation of ACS14 into the nanoparticle matrix. Energy‐dispersive X‐ray spectroscopy (EDX) further confirmed the presence of carbon (C), oxygen (O), and sulfur (S) within the particles (Figure 2E), validating the encapsulation of ACS14. In addition, the drug loading and encapsulation efficiency of ACS14@BSA were determined to be 10.2 ± 1.3% and 37.8 ± 5.3%, respectively (Table S1). ACS14@BSA nanoparticles were prepared via protein denaturation followed by crosslinking with glutaraldehyde through Schiff base bonds, which are intrinsically pH‐sensitive [45]. This design enables the responsive release of H_2_S and aspirin within acidic intracellular compartments. Accordingly, we evaluated the release profile of ACS14@BSA under acidic conditions (pH 5.5) that mimic the late endosomal/lysosomal environment [46, 47]. As shown in Figure 2F,G, ACS14@BSA released approximately 116.6 µm H_2_S and 93.1% of aspirin within 12 h, approaching maximal release. These results indicate that ACS14@BSA exhibits favorable pH‐responsive and sustained release behavior.

In our previous study, we demonstrated that BSA nanoparticles can specifically target endometriotic lesions via a neutrophil hitchhiking strategy [6]. Building on this finding, we next examined whether ACS14‐loaded BSA nanoparticles could also specifically target endometriotic lesions. Fluorescein isothiocyanate (FITC) labeling was performed on ACS14@BSA nanoparticles to obtain FITC‐ACS14@BSA. Endometriosis model mice were intraperitoneally injected with either FITC‐ACS14@BSA nanoparticles or free FITC dye as a control. Sixteen hours after injection, fluorescence distribution across various tissues was assessed. As shown in Figure 2H,I, FITC‐ACS14@BSA exhibited preferential accumulation in ectopic lesions, with significantly higher fluorescence intensity compared to eutopic endometrium. In contrast, free FITC showed no significant difference in fluorescence between ectopic and eutopic sites (Figure 2H,I). In addition, the fluorescence intensity of FITC‐ACS14@BSA in ectopic lesions was significantly higher than that in the free FITC group (Figure 2I), with a significantly increased ectopic‐to‐eutopic fluorescence intensity ratio compared with the free FITC group (Figure 2J). Quantitative analysis further revealed that the fluorescence intensities in major organs, including the heart, liver, spleen, lungs, and kidneys, were not significantly different between the FITC‐ACS14@BSA and free FITC groups. (Figure 2K). Collectively, these results suggest that ACS14@BSA enables specific targeted delivery to endometriotic lesions.

### 2.2 In Vivo Therapeutic Efficacy and Underlying Mechanisms of ACS14@BSA in Endometriosis

**FIGURE 3:**
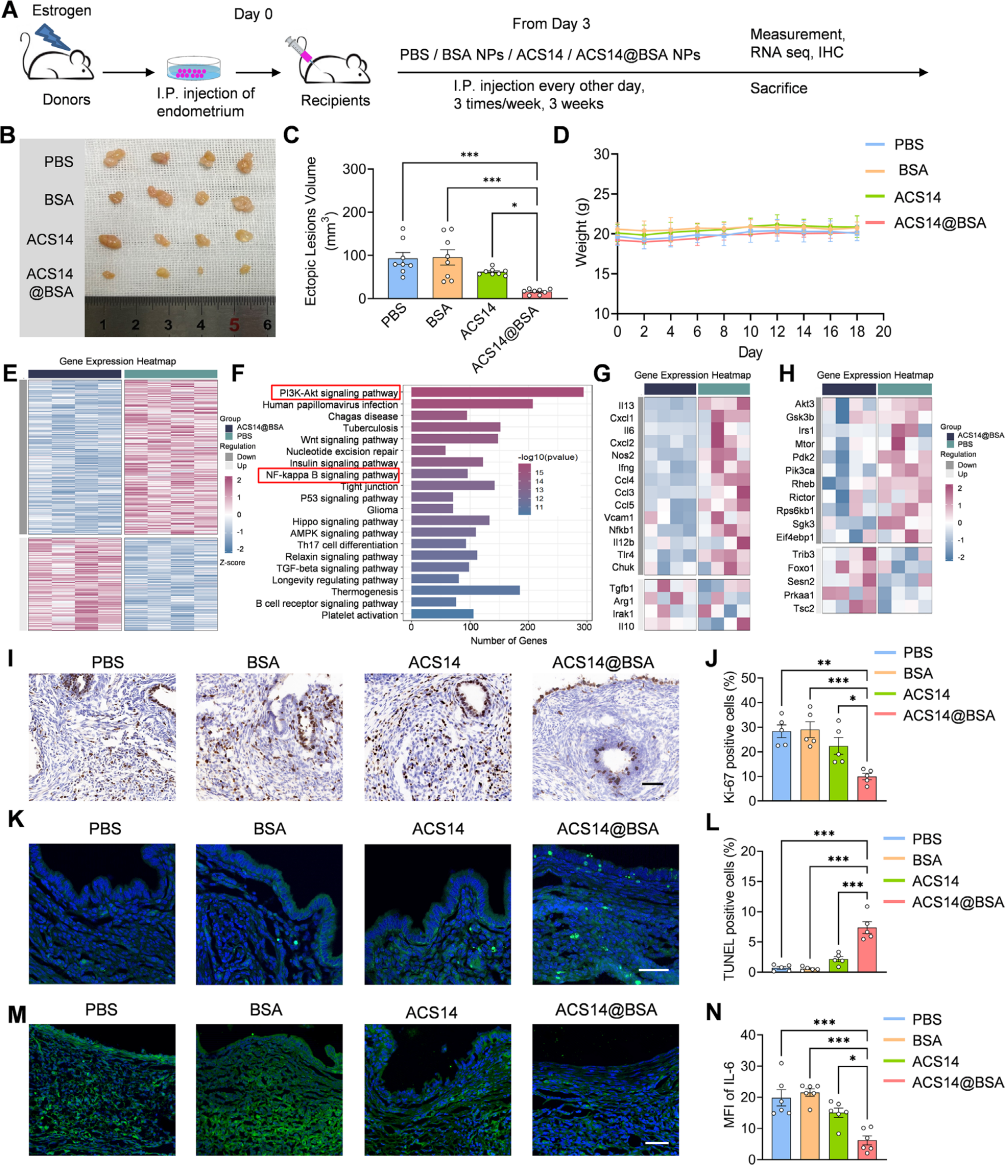
In vivo anti‐endometriosis efficacy of ACS14@BSA. (A) Schematic drawing of treatment with different formulations, starting on day 3. (B) Images and (C) total volume of endometriotic lesions in mice following different treatments (*n* = 8). (D) Monitoring the body weight of mice receiving different treatments (*n* = 4). (E–H) RNA‐seq analysis reveals the differentially expressed genes (DEGs) in murine lesion tissues from two groups: ACS14@BSA and PBS (*n* = 4). (E) Heatmap showing the significantly regulated genes identified in RNA‐seq analysis of murine ectopic lesion tissue. Gene expression values were normalized. (F) KEGG pathway enrichment analysis comparing the murine treated with ACS14@BSA to those treated with PBS. (G) Heatmap representing the genes involved in the NF‐κB signaling pathway detected in RNA‐seq analysis of murine ectopic lesion tissues. (H) Heatmap representing the genes involved in the PI3K/Akt signaling pathway detected in RNA‐seq analysis of murine ectopic lesion tissues. (I) Ki67 staining of ectopic lesion tissues. Scale bar, 50 µm. (J) Quantification of Ki67‐positive cells in ectopic lesion tissues (*n* = 5). (K) Fluorescence images of TUNEL staining in endometriotic lesions. Blue staining is DAPI, green staining is TUNEL. Scale bar, 50 µm. (L) Quantification of TUNEL‐positive cells in ectopic lesion tissues (*n* = 5). (M) IL‐6 staining of ectopic lesion tissues. Blue staining is DAPI, green staining is IL‐6. Scale bar, 50 µm. (N) Quantification of IL‐6 mean fluorescence intensity (MFI) in ectopic lesions (*n* = 6). Data are shown as the mean ± SEM. ^*^ *p* < 0.05, ^**^ *p* < 0.01, ^***^ *p* < 0.001.

To evaluate the therapeutic efficacy of ACS14@BSA in endometriosis, a minimally invasive mouse model was established by transplanting minced endometrial fragments from donor mice into the peritoneal cavity of syngeneic recipient mice, following established protocols [48, 49] (Figure 3A). The recipient mice were then randomly assigned to four groups to assess the therapeutic efficacy of different formulations (Figure 3A). As shown in Figure 3B, after intraperitoneal injection of ACS14@BSA, a significant reduction in the size of ectopic endometrial lesions was observed. Statistical analysis confirmed that lesion volumes in the ACS14@BSA‐treated group were significantly smaller than those in the PBS and BSA treatment groups (Figure 3C). Notably, the ACS14@BSA group showed significantly smaller lesion volumes than the free ACS14 group (Figure 3B,C), highlighting the advantages of BSA nanoparticles in enhancing drug delivery and therapeutic outcomes. In addition, throughout the treatment period, no significant changes in body weight were observed across the groups, indicating good systemic tolerance of ACS14@BSA. (Figure 3D).

Subsequently, to better understand the therapeutic mechanism of ACS14@BSA, we performed RNA sequencing analysis on endometriotic lesion tissues from the ACS14@BSA‐treated group and the control group (PBS group) in mice. Differential gene expression analysis (Figure 3E) revealed significant transcriptional changes between the two groups. KEGG pathway enrichment analysis (Figure 3F) indicated that the differentially expressed genes were significantly enriched in several key signaling pathways, including the PI3K/Akt and NF‐κB pathways. Notably, genes associated with these pathways showed a coordinated overall alteration in the ACS14@BSA group (Figure 3G,H), suggesting that ACS14@BSA may exert its therapeutic effects by modulating these signaling cascades. The PI3K/Akt pathway is critical for regulating cell survival and proliferation, and H_2_S has been reported to inhibit this pathway [25, 26]. To further investigate the effect of ACS14@BSA on lesion cell viability, we assessed cell proliferation and apoptosis in ectopic endometrial tissues. As shown in Figure 3I–L, immunohistochemistry and immunofluorescence staining demonstrated a significant reduction in Ki67‐positive proliferating cells and a marked increase in TUNEL‐positive apoptotic cells in the ACS14@BSA group compared to other groups. These results indicate that ACS14@BSA effectively suppresses cell proliferation and promotes apoptosis within endometriotic lesions. Moreover, the NF‐κB signaling pathway is closely linked to inflammation, and aspirin is known to suppress this pathway [27, 28, 29]. Our data revealed that the expression levels of pro‐inflammatory cytokines IL‐6 was significantly reduced in the ACS14@BSA group compared to other groups (Figure 3M,N), supporting a strong anti‐inflammatory effect. Taken together, these results indicate that ACS14@BSA reduces endometriotic lesion progression via dual mechanisms—H_2_S‐mediated PI3K/Akt inhibition and aspirin‐induced suppression of NF‐κB–driven inflammation.

Of note, hemolysis assays, serum biochemical analyses, complete blood counts, and H&E staining of major organs indicated no red blood cell lysis or histopathological abnormalities, confirming the excellent biosafety of the ACS14@BSA formulation (Figures S2–S4). These results collectively highlight ACS14@BSA as a promising therapeutic platform for the treatment of endometriosis, offering both efficacy and safety.

### 2.3 Transport of H_2_S Released From ACS14@BSA at Ectopic Lesions to the Anterior Cingulate Cortex in Brain

**FIGURE 4:**
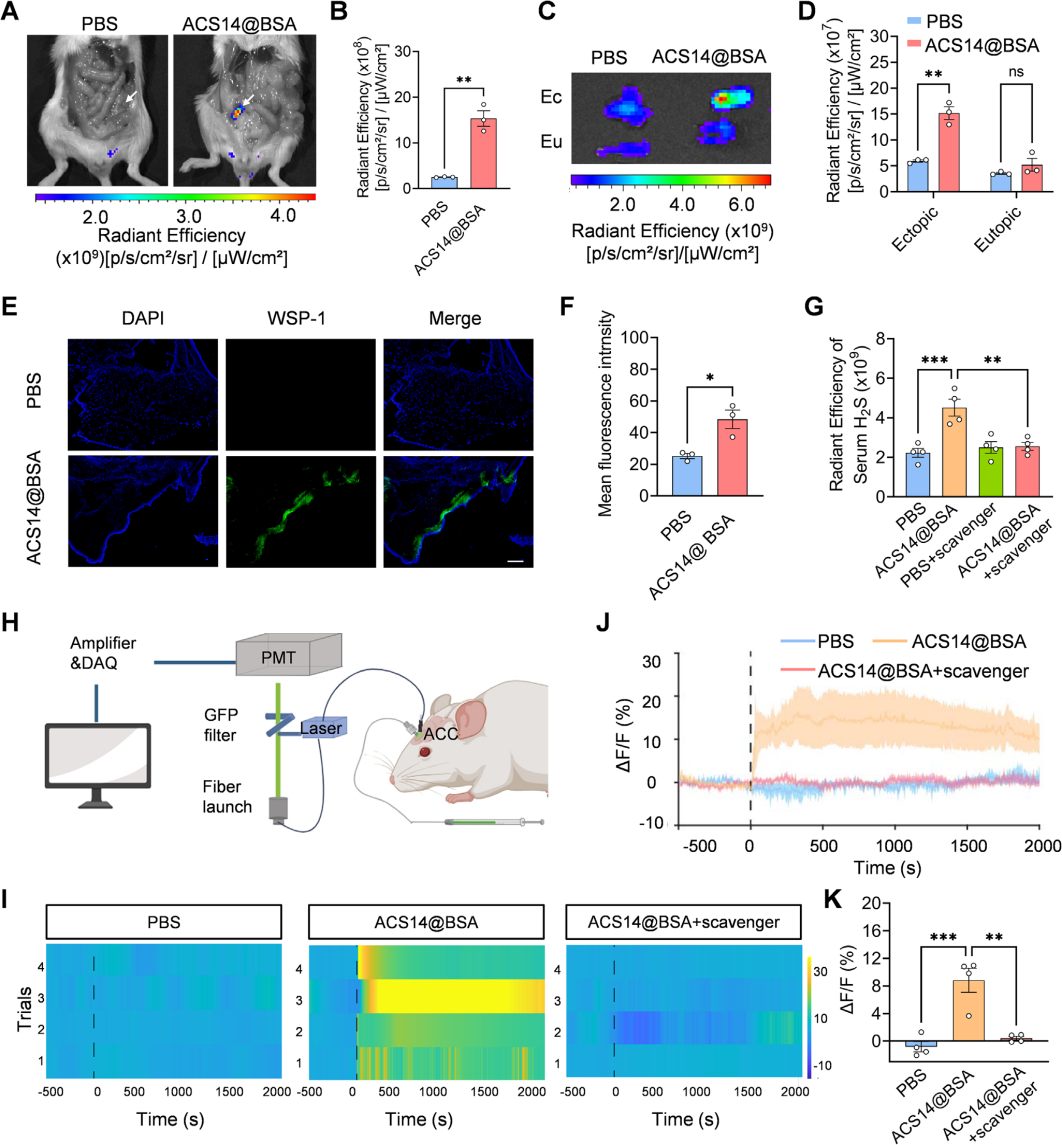
Validating bloodstream delivery of H_2_S from ectopic lesions to the ACC. (A) In vivo imaging of H_2_S levels in endometriosis mice assessed by a fluorescent probe WSP‐1. Arrows denote ectopic endometriotic lesions. (B) Quantitative fluorescence intensity of ectopic lesions from (A) (*n* = 3). (C) Ex vivo imaging of H_2_S levels in ectopic lesions and eutopic endometrium assessed by a fluorescent probe WSP‐1. Ec, ectopic lesion; Eu, eutopic endometrium. (D) Quantitative fluorescence intensity of ectopic lesions and eutopic endometrium from (C). (E) Fluorescent images of frozen sections of ectopic lesions and (F) their statistical results (*n* = 3). (G) Serum levels of H_2_S in mice following various treatments (*n* = 4). (H) Schematic of fiber photometry and cannula administration in endometriosis mice. (I) Heatmaps, (J) time‐course curves, and (K) quantification of alterations in H_2_S signals within the ACC of mice following different treatments (*n* = 4). Data are shown as the mean ± SEM. ^*^ *p* < 0.05, ^**^ *p* < 0.01, ^***^ *p* < 0.001; ns, not significant.

In addition to its anti‐endometriotic properties, H_2_S has been reported to exert rapid anxiolytic effects [50]. As a multifunctional gaseous neurotransmitter, H_2_S is implicated in the pathophysiology of anxiety‐related behaviors [51], importantly, can readily cross the blood–brain barrier (BBB). Based on these characteristics, we investigated the ectopic lesions‐to‐brain transport of H_2_S released from ACS14@BSA. First, following intraperitoneal injection of ACS14@BSA, H_2_S in ectopic lesions and eutopic endometrium was visualized using Washington State Probe‐1 (WSP‐1) as a bright green fluorescent signal [52, 53]. As shown in Figure 4A–D, strong fluorescence was observed in ectopic lesions in the ACS14@BSA‐treated group, whereas only negligible signals were detected in eutopic endometrium, indicating lesion‐specific H_2_S release. Consistently, frozen‑section imaging revealed intense fluorescence in the ectopic lesions of ACS14@BSA‑treated mice (Figure 4E,F). Together with our earlier data demonstrating that ACS14@BSA enables specific targeted delivery to endometriotic lesions, and prior evidence that BSA nanoparticles are formed via protein denaturation followed by glutaraldehyde crosslinking through Schiff‐base bonds that are intrinsically pH‐sensitive [6, 42], these findings support that intraperitoneally administered ACS14@BSA nanoparticles achieve lesion‐targeted delivery and localized release of H_2_S in ectopic endometrial lesions.

Considering that pain is a major driver of anxiety in endometriosis [11, 36] and that the anterior cingulate cortex (ACC) is a key hub for pain–affect integration [37, 38], we investigated whether H_2_S released from ACS14@BSA could reach the ACC and alleviate anxiety. Given the rich vascularization of endometriotic lesions, the elevated H_2_S levels in the bloodstream, and the ability of H_2_S to cross the blood–brain barrier, we hypothesized that a fraction of the H_2_S released at lesion sites could be transported to the ACC via the circulation. To test this hypothesis, we first measured serum H_2_S levels in mice after intraperitoneal administration of ACS14@BSA and observed a marked elevation in circulating H_2_S (Figure 4G). We then employed a fiber photometry system combined with H_2_S fluorescent probes for real‐time and sensitive detection of H_2_S fluctuations in the ACC of living mice (Figure 4H) [21]. The results showed that ACS14@BSA administration led to a significant increase in fluorescent signal in the ACC of treated mice compared to control mice, demonstrating elevated levels of H_2_S in the ACC (Figure 4I–K). We then investigated whether H_2_S was delivered to the ACC via the bloodstream. To test this, we pre‐injected hydroxocobalamin—a H_2_S scavenger that does not easily cross the blood–brain barrier [54, 55]—into the tail vein to eliminate circulating H_2_S. As shown in Figure 4G, hydroxocobalamin pre‐treatment abolished the increase in blood H_2_S levels following ACS14@BSA administration. Consequently, no elevation in H_2_S levels was detected in the ACC (Figure 4I‐K), supporting the hypothesis that H_2_S released from ACS14@BSA reaches the ACC via blood‐mediated transport.

### 2.4 ACS14@BSA Alleviate the Hyperactivation of Glutamatergic Neurons in the Anterior Cingulate Cortex

**FIGURE 5:**
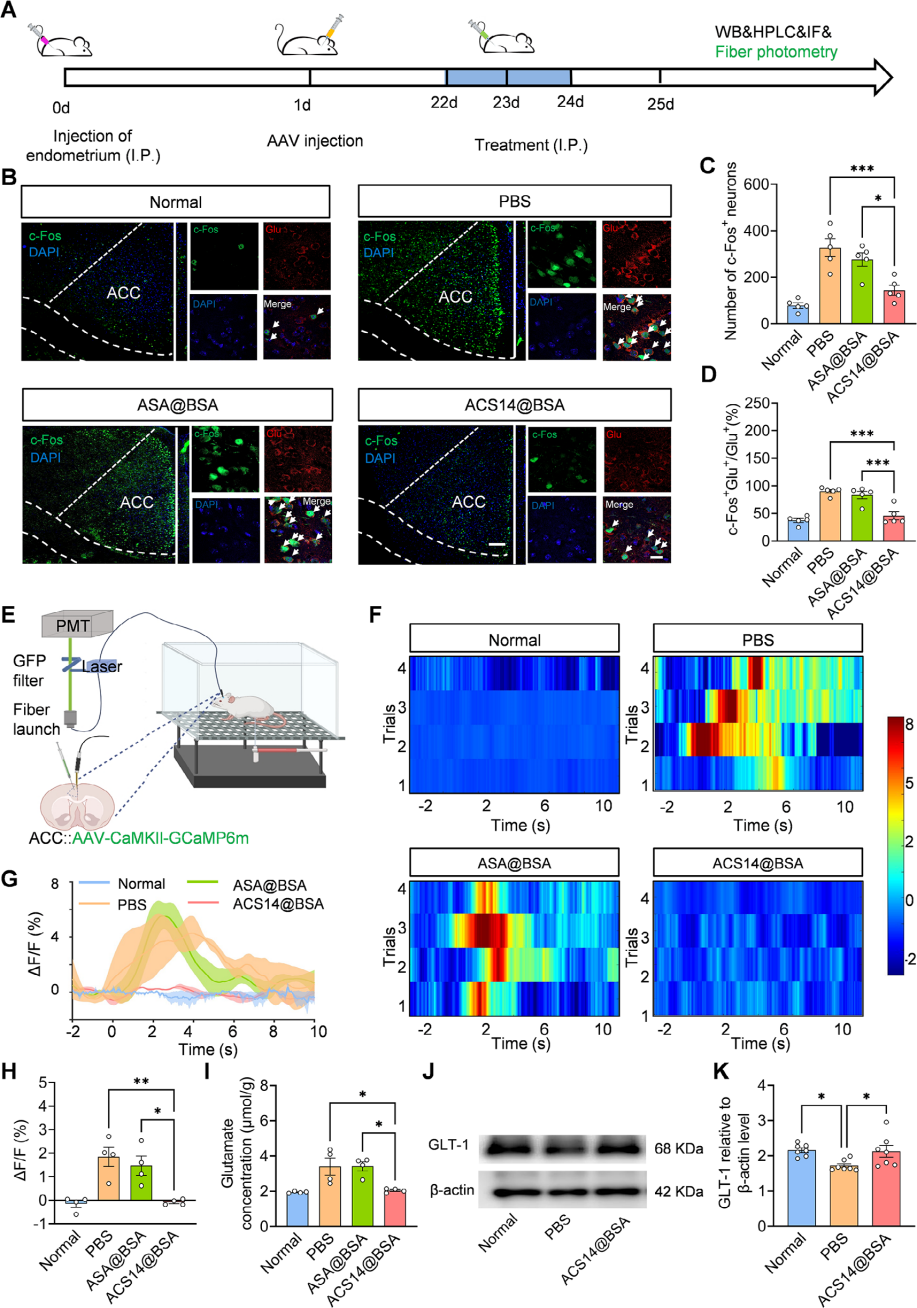
ACS14@BSA restores Glu^ACC^ neuronal activity by upregulating GLT‐1 and reducing glutamatergic hyperactivation in the ACC. (A) Experimental scheme of modeling and drug treatments, as well as western blotting, high‐performance liquid chromatography, immunofluorescence, and fiber photometry recording. (B) Representative IF images of c‐Fos and glutamate staining in the ACC. Scale bar: 200 µm (left) and 20 µm (right). (C) Quantification of c‐Fos–positive neurons in the ACC(*n* = 5). (D) Summary data showing the percentage of glutamate‐positive neurons expressing c‐Fos in the ACC (*n* = 5). (E) Schematic of fiber photometry for detecting Glu^ACC^ activity. (F) Heatmaps, (G) time‐course curves, and (H) quantification of alterations in GCaMP6m signals within the ACC of mice following different treatments (*n* = 4). (I) HPLC quantification of glutamate levels in the ACC (*n* = 4). (J,K) Western blotting of GLT‐1 level in ACC (*n* = 4). Data are shown as the mean ± SEM. ^*^ *p* < 0.05, ^**^ *p* < 0.01, ^***^ *p* < 0.001. Normal group: mice without endometriosis modeling that received intraperitoneal PBS.

Glutamatergic neurons, the dominant excitatory cell type within the ACC, are known to be closely involved in the regulation of anxiety‐related behaviors [38, 39, 40]. Based on this understanding, we proposed that H_2_S might influence the function of glutamatergic neurons in the ACC (Glu^ACC^) and thereby produce anxiety‐reducing effects [21]. To test this hypothesis, animals were divided into four groups: a normal group, a PBS group (endometriosis mice treated with PBS), an ASA@BSA‐treated group (endometriosis mice treated with aspirin‐loaded BSA nanoparticles), and an ACS14@BSA‐treated group (endometriosis mice treated with ACS14@BSA nanoparticles). Notably, the inclusion of the ASA@BSA group served to distinguish the specific regulatory effects of H_2_S from those of aspirin alone. Subsequently, to assess the functional status of Glu^ACC^ neurons, we employed co‐immunofluorescence staining targeting both c‐Fos and glutamate (Figure 5A). As observed in Figure 5B,C, mice in the PBS group showed a considerable increase in c‐Fos‐positive cells within the ACC when compared to the normal group, suggesting heightened neuronal activation. This activation was notably reduced in the ACS14@BSA‐treated group. A parallel trend was seen in c‐Fos‐positive glutamatergic neurons (Figure 5B,D), which supports the conclusion that ACS14@BSA treatment can attenuate Glu^ACC^ hyperexcitability triggered by endometriosis. Notably, ASA@BSA treatment did not reduce the number of c‐Fos‐positive or c‐Fos‐positive glutamatergic neurons compared to PBS‐treated mice (Figure 5B–D), highlighting the specific role of H_2_S in diminishing Glu^ACC^ neurons hyperactivity.

To strengthen the experimental conclusions, we applied in vivo calcium imaging using fiber photometry to monitor neuronal responses (Figure 5E) [56, 57]. This approach enabled real‐time observation of calcium fluctuations in Glu^ACC^ neurons, which serve as a key proxy for neuronal excitability. For selective calcium signal tracking, mice were injected with AAV‐CaMKIIα‐GCaMP6m, enabling the expression of the fluorescent calcium indicator GCaMP6m specifically under the control of the CaMKIIα promoter [58]. The virus was stereotaxically injected into the ACC, leading to calcium indicator expression in glutamatergic neurons after three weeks. In freely moving mice, PBS‐treated endometriosis mice exhibited a marked elevation in calcium activity within Glu^ACC^ neurons following tactile stimulation of the abdominal surface using 0.07 g von Frey filaments. In contrast, ACS14@BSA‐treated mice demonstrated a clear attenuation of this calcium response compared with PBS‐treated controls (Figure 5F–H). Collectively, these observations support the conclusion that ACS14@BSA can dampen the excessive activation of Glu^ACC^ neurons that is associated with endometriosis‐induced sensitization. We further investigated the mechanism by which ACS14@BSA mitigates the hyperactivity of Glu^ACC^ neurons. Glutamate, the most prevalent excitatory neurotransmitter in the central nervous system, has the capability to activate glutamatergic neurons [59, 60]. Subsequently, glutamate concentrations in the ACC were quantified using HPLC. As shown in Figure 5I, mice in both the PBS and ASA@BSA‐treated groups exhibited significantly elevated glutamate levels in the ACC. In contrast, treatment with ACS14@BSA effectively reversed this increase, restoring glutamate concentrations to near‐normal levels (Figure 5I). Glutamate transporters (GLT), responsible for over 90% of glutamate uptake in adult brain is crucial for maintaining glutamate homeostasis and preventing glutamate toxicity [61, 62]. Previous studies have demonstrated that exogenous H_2_S, due to its reductive properties, can upregulate GLT‐1 expression and augment extracellular glutamate uptake by astrocytes [63, 64]. In this study, we observed a reduction in GLT‐1 levels in the ACC of PBS‐treated mice, whereas mice treated with ACS14@BSA showed an upregulation of GLT‐1 compared to the PBS group (Figure 5J,K). These findings suggest that the restoration of normal activity in Glu^ACC^ neurons by ACS14@BSA may be attributed to upregulation of GLT‐1, which enhances extracellular glutamate uptake and helps maintain glutamate homeostasis.

### 2.5 ACS14@BSA Alleviates Anxiety‐Like Behaviors in Endometriosis Mice

**FIGURE 6:**
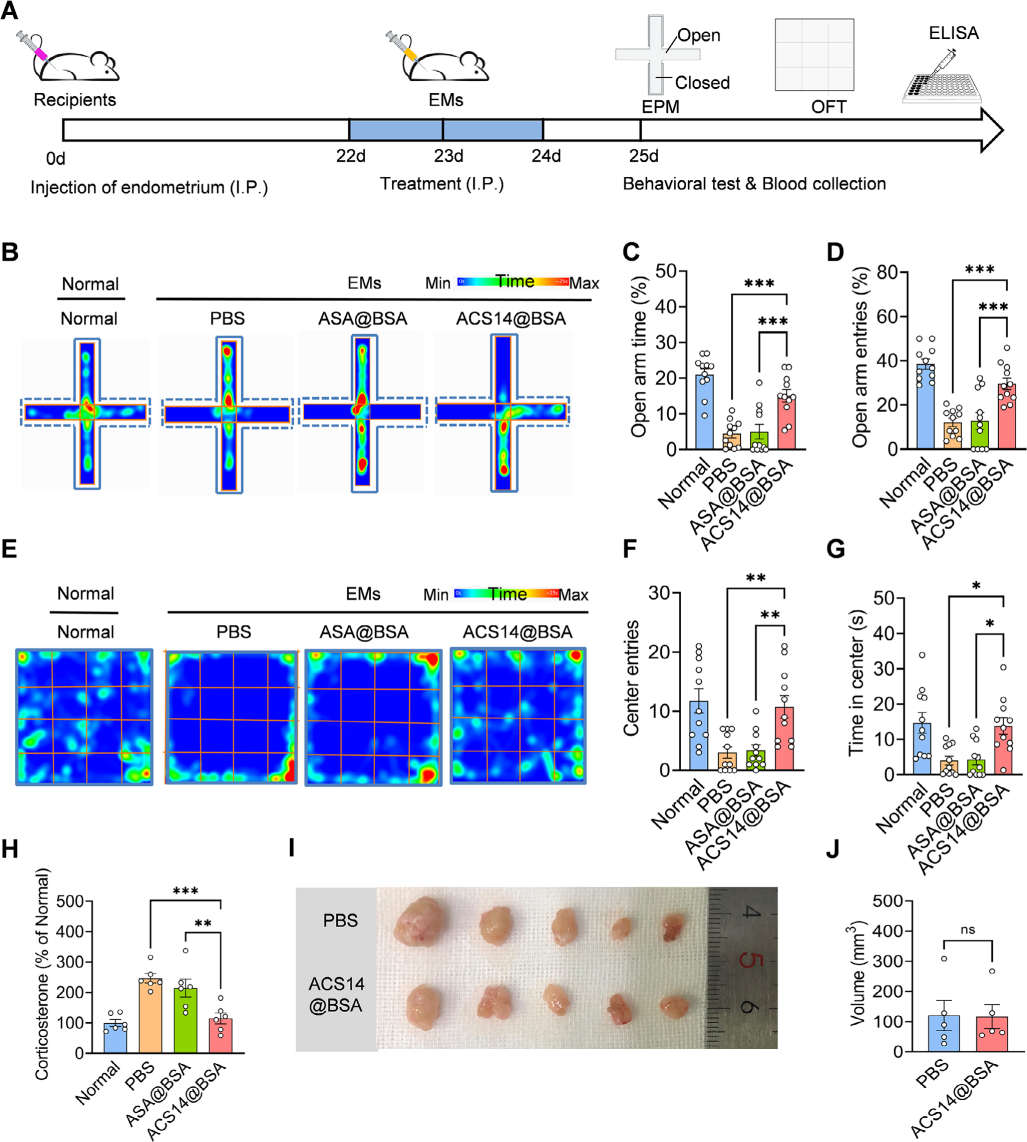
ACS14@BSA relieves anxiety‐like behaviors in endometriosis mice. (A) Experimental scheme of modeling and drug treatments, as well as timeline of subsequent behavioral tests and blood collection. (B) Tracing of locomotion for representative animals in EPM. (C) Percentage of time spent in the open arms of EPM. (D) Percentage of entry numbers in the open arms of EPM. (E) Tracing of locomotion for representative animals in OFT. (F) Number of central zone entries in OFT. (G) Time of central zone spent in OFT, *n* = 11. (H) Relative serum corticosterone levels (*n* = 6). (I) Images and (J) total volume of endometriotic lesions in mice following different treatments, *n* = 5. Data are shown as the mean ± SEM. ^*^ *p* < 0.05, ^**^ *p* < 0.01, ^***^ *p* < 0.001; ns, not significant. OFT, open field test; EPM, elevated plus maze.

Excessive activation of Glu^ACC^ neurons has been linked to anxiety‐related phenotypes, whereas ACS14@BSA has been demonstrated to normalize their hyperactivity. Building upon these observations, we investigated whether ACS14@BSA can also attenuate anxiety in mice associated with endometriosis. Three weeks post‐model induction, we conducted a comprehensive behavioral evaluation using the elevated plus maze (EPM) and open field test (OFT)—two established paradigms for assessing rodent anxiety (Figure 6A). Compared with normal mice, PBS‐treated endometriosis mice spent a markedly reduced proportion of time in the EPM's open arms and made fewer open‐arm entries (Figure 6B–D). A similar pattern emerged in the OFT, where they exhibited fewer entries and less time exploring the center zone (Figure 6E–G), collectively indicating significant anxiety‐like behaviors. In parallel, we quantified serum corticosterone—an established biomarker of stress responses [65]. Endometriosis PBS‐treated mice exhibited higher corticosterone levels than normal mice (Figure 6H). However, ACS14@BSA treatment led to an amelioration of anxiety‐like behaviors (Figure 6B–G) alongside a pronounced reduction in serum corticosterone (Figure 6H). Notably, ASA@BSA failed to mitigate either the elevated corticosterone or the behavioral manifestations (Figure 6B–H), suggesting that the anxiolytic effect is specifically attributable to H_2_S released by ACS14@BSA. Additionally, locomotor activity, measured by total distance traveled in both the EPM and OFT, showed no group differences (Figure S5), ruling out confounding effects from motor impairments. Importantly, repeated ACS14@BSA administration over three consecutive days did not influence lesion size compared with PBS groups (Figure 6I,J), implying that the anxiolytic effect operates independently of lesion progression. Collectively, these results demonstrate that ACS14@BSA elicits notable anxiolytic effects in endometriosis mice, independent of its therapeutic effects on ectopic lesions.

## 3 Conclusion

In summary, we have proposed a novel gasotransmitter‐based strategy that offers a promising non‐invasive, non‐hormonal approach for the simultaneous treatment of endometriosis and its comorbid anxiety. We developed ACS14@BSA nanoparticles by loading ACS14—an H_2_S‐releasing aspirin derivative—into BSA nanoparticles to enable controlled H_2_S and aspirin release. This formulation inhibits endometriosis growth through the combined anti‐proliferative and anti‐inflammatory effects of H_2_S and aspirin, respectively. Furthermore, ACS14@BSA targets ectopic lesions, where the released H_2_S is transported through the bloodstream to the anterior cingulate cortex (ACC) in the brain. In the ACC, H_2_S reduces glutamatergic neuron hyperactivity, thereby alleviating anxiety‐like behaviors. Our strategy employs BSA nanoparticles as a carrier, offering translational potential and therapeutic benefits for endometriosis by targeting both physiological and psychological aspects to improve prognosis.

## 4 Experimental Section

### 4.1 Synthesis of ACS14@BSA NPs

The BSA nanoparticles were synthesized using a simple, low‑cost, and reproducible method [6]. 10 mg of BSA (EZ652D3714, BioFROXX) was dissolved in 1 mL of deionized water and stirred until fully dissolved. Subsequently, 3.0 mg of ACS14 (YSZC3349, Jilin Chinese Academy of Sciences—Yanshen Technology Co., Ltd.) powder was dissolved in 100 µL of DMSO (ST038, Beyotime) under stirring until complete dissolution. The ACS14 solution was then thoroughly mixed with the BSA aqueous solution in a clean glass vial. The mixture was stirred at room temperature for 1 h at 600 rpm, followed by the dropwise addition of 3 mL absolute ethanol, resulting in an observable turbidity indicating nanoparticle formation. To stabilize the formed nanoparticles, 10 µL of 2.5% glutaraldehyde (G105905, Aladdin) was added for cross‐linking BSA molecules, followed by further stirring at 600 rpm for 6 h at room temperature. The solution was then centrifuged at 12 000 rpm and 4°C for 10 min. To remove residual organic solvents from the precipitate, the pellet was resuspended in ultrapure water, vortexed, and centrifuged again; this washing step was repeated three times. Finally, the obtained nanoparticles were dispersed in 1 mL PBS solution for further use. BSA NPs were prepared in parallel using the same procedure, except that ACS14 was omitted. ASA@BSA NPs were prepared using the same procedure, with ACS14 replaced by aspirin, while all other conditions were kept identical.

### 4.2 Synthesis of FITC‐ACS14@BSA NPs

Dissolve 10 mg of BSA and 1 mg of FITC (HY‐66019, MCE) in 5 mL of 100 mM sodium bicarbonate buffer (pH 8.5) and incubate at room temperature for 24 h. Excess FITC was removed using a 50 kDa ultrafiltration tube. Then, 1 mL of BSA solution (10 mg/mL) was mixed with 100 µL of BSA‐FITC solution (10 mg/mL), followed by the addition of 3 mg ACS14, and the mixture was stirred at 600 rpm for 1 h. Afterward, 3 mL of absolute ethanol was added dropwise, and 10 µL of a 2.5% glutaraldehyde solution was added 1 h later, followed by further stirring for 6 h. The solution was then centrifuged at 12 000 rpm and 4°C for 10 min, and the pellet was resuspended in ultrapure water, vortexed, and centrifuged again. This washing step was repeated three times to prepare the FITC‐ACS14@BSA nanoparticles. To prepare free FITC solution as a control, 1 mg of FITC was dissolved in PBS.

### 4.3 Characterization of ACS14@BSA NPs

The morphology of ACS14@BSA NPs and BSA NPs was characterized using a transmission electron microscope (JEM‐2010). The elemental composition of ACS14@BSA NPs was analyzed by EDX (Oxford tsr). The hydrodynamic size of ACS14@BSA NPs was measured using DLS with a particle size analyzer (Zetasizer Nano ZS, Malvern). Additionally, the absorption characteristics of ACS14@BSA NPs, BSA NPs, and ACS14 powder under infrared radiation at different wavelengths were examined using FTIR (Nicolet iS50, Thermo Scientific).

### 4.4 Drug Encapsulation of ACS14@BSA NPs

Following the glutaraldehyde crosslinking and centrifugation steps during the synthesis of ACS14@BSA nanoparticles, the supernatant was collected. The absorbance of the supernatant at 276 nm was measured using a microplate reader (Infinite M200). The amount of free ACS14 in the supernatant was calculated based on a standard calibration curve. Using the initial drug input (3.0 mg), the drug encapsulation efficiency and loading capacity of the BSA nanoparticles were subsequently determined.

### 4.5 In Vitro Drug Release

One milliliter of ACS14@BSA NP solution was placed into a glass vial, and then 4 mL of PBS, adjusted to pH 5.5 and pre‐warmed to 37°C was gently added. The vial was incubated in a thermostatic shaker at 37°C and gently shaken at 70 rpm. At predetermined intervals (0, 2, 4, 6, 12, 24, and 48 h), 1 mL of the mixed solution was withdrawn and replenished with 1 mL of fresh ultrapure water at 37°C. Finally, the absorbance at 276 nm of each collected sample was measured using a Micro UV–vis spectrophotometer (LIFEREAL, FC‐1100) to determine aspirin concentration based on the standard calibration curve. The concentration of released H_2_S was measured using an H_2_S Content Assay Kit (BC2055, Solarbio).

### 4.6 Mice

Female Balb/c mice, aged six to eight weeks, weighing 18 to 25 g, were purchased from GemPharmatech Co., Ltd. The mice were kept in controlled environments with regulated temperature, humidity, and lighting, and were provided with unrestricted access to water. All procedures involving the animals complied with the Ethical Regulations on the Care and Use of Laboratory Animals established by Anhui Medical University and received approval from the university's animal experiment committee.

### 4.7 Model of Endometriosis

Mice were divided into donor and recipient groups. Donor mice received a single subcutaneous injection of estradiol benzoate (3 µg per mouse; MCE). One week after the injection, the donor mice were euthanized, and their uterine horns were collected. The uterine horns were longitudinally incised using surgical scissors and carefully cut into small tissue fragments. Fragments from the uterine horns of three mice were intraperitoneally injected into four recipient mice to induce endometriosis.

### 4.8 Distribution of ACS14@BSA NPs in Mice

Endometriosis model mice were intraperitoneally injected with 50 µL of FITC‐labeled ACS14@BSA nanoparticles (FITC‐ACS14@BSA NPs). After 16 h, the ectopic lesions, eutopic endometrium, and major organs including the heart, liver, spleen, lungs, and kidneys were harvested. Fluorescent images were acquired using an in vivo imaging system (IVIS, PerkinElmer) to analyze the tissue distribution of FITC@BSA NPs. As a control, 50 µL of free FITC solution was intraperitoneally injected into endometriosis mice. After 16 h, tissues were collected and imaged using the same IVIS system.

### 4.9 In Vivo Therapy

The endometriosis model mice were randomly divided into four groups. Starting from day 3 after the injection of donor endometrial fragments, each group was intraperitoneally injected every other day with 100 µL of PBS, BSA‐NPs, ACS14, and ACS14@BSA. Body weight was measured every other day. After 3 weeks, mice were individually euthanized, and lesions were excised and processed for disease evaluation or immunohistochemical staining. Lesions were measured with calipers, and the extent of endometriosis was assessed by calculating the total volume of all lesions in each mouse. The volume of each lesion was determined using the formula: 0.5 × length × width^2^.

### 4.10 mRNA Sequencing

mRNA isolation, library preparation, sequencing, and differential expression analysis were conducted by OE Biotech Co., Ltd. (Shanghai, China). Total RNA from the BSA group and ACS14@BSA group was extracted using TRIzol reagent according to the manufacturer's protocol. RNA purity and quantity were assessed using a NanoDrop 2000 spectrophotometer (Thermo Scientific), while RNA integrity was evaluated using an Agilent 2100 Bioanalyzer (Agilent Technologies, Santa Clara). Transcriptome libraries were prepared using the VAHTS Universal V6 RNA‐seq Library Prep kit as per the manufacturer's instructions. The Illumina NovaSeq 6000 platform was employed to sequence the libraries, producing 150 bp paired‐end reads (∼50 million reads/sample). Initial processing of raw reads utilized fastp software to eliminate low‐quality reads, resulting in approximately 48 million clean reads. These reads underwent mapping to the reference genome using a hierarchical index tailored for transcript splicing comparisons. Gene expression levels were quantified using exon per kilobase/million mapped fragments (FPKM), with read counts determined by HTSeq‐count. Principal component analysis (PCA) was conducted in R (v3.2.0) to evaluate the biological consistency across samples. Differentially expressed gene (DEG) analysis was performed using the DESeq2 package. Genes with a q‐value < 0.05 and a fold change > 2 were defined as DEGs. Hierarchical clustering analysis of DEGs was conducted using R (v3.2.0) to visualize expression patterns across different groups and samples. Subsequently, KEGG pathway enrichment analysis of the DEGs was performed based on the hypergeometric distribution algorithm to identify significantly enriched pathways. Bar plots of the significantly enriched pathways were generated using R (v3.2.0). Based on the KEGG pathway enrichment results, we further selected two significantly enriched pathways relevant to this study, extracted the DEGs involved in these pathways, and performed hierarchical clustering in R (v3.2.0) using the normalized expression matrix. A heatmap was then generated to visualize the expression patterns across different groups.

### 4.11 Histological Analyses

Immunohistochemistry for Ki67 (GB111499‐100, Servicebio), immunofluorescence for IL‐6 (ab290735, abcam) and TNF‐*α* (17590‐1‐AP, Proteintech), and H&E staining were performed on paraffin‐embedded mouse tissue sections. To detect apoptosis in the endometriosis lesions, additional frozen sections were prepared and TUNEL assay (C1088, Beyotime) was performed according to the manufacturer's instructions. All sections were scanned using a confocal microscope (LSM880, Zeiss) or Olympus IX71 fluorescence microscope. Positive‐stained cells were counted in four randomly selected fields, and the staining signals were quantified.

### 4.12 Stereotaxic Viral Injection

Mice were anesthetized with an intraperitoneal injection of pentobarbital sodium (20 mg/kg) and secured in a stereotaxic apparatus (RWD Life Science). Core body temperature was maintained at 36°C using a heating pad throughout the procedure. After a midline incision of the scalp to expose the skull, the skull was leveled, and small holes were drilled using a dental drill. A glass micropipette loaded with the designated virus was connected to a microsyringe pump (RWD Life Science) for infusion. Viral volumes ranging from 200 to 300 nL were delivered at a constant rate of 50 nL /min, adjusted based on viral titer and expression efficiency. The anterior cingulate cortex (ACC) was targeted using the following stereotaxic coordinates: anteroposterior (AP) +1.09 mm from bregma, mediolateral (ML) ±0.3 mm from the midline, and dorsoventral (DV) −1.75 mm from the cortical surface. To minimize viral reflux, the injection pipette was left in place for an additional 10 min after infusion.

For fiber‐photometry recordings, mice received contralateral stereotaxic injections into the ACC of 300 nL AAV2/9‐CaMKII*α*‐GCaMP6m (PT‐0111, BrainVTA) or AAV2/9‐hSyn‐iGluSnFR (PT‐1140, BrainVTA) at AP +1.09 mm, ML +0.30 mm, DV −1.75 mm to report Ca^2^^+^ signals or glutamate levels, respectively. Immediately after virus delivery, an optical fiber (200 µm OD, NA 0.39; RWD Life Science) mounted on a cannula was positioned with its tip 20 µm dorsal to the injection site and fixed using Super‐Bond C2B dental acrylic (NISSIN, Yokohama). Photometry was performed no earlier than two weeks after injection.

For cannula infusion, a single guide cannula (RWD Life Science) was implanted above the ACC. An optical fiber was inserted at an oblique angle beneath the cannula to monitor fluorescence signals. Following a postoperative recovery period of no less than 14 days, a hydrogen sulfide (H_2_S) probe (WSP‐1, 1 mM; Shanghai Maokang Biotechnology Co. Ltd.) was administered via a microinjector cannula, which extended 0.5 mm beyond the guide cannula tip.

### 4.13 In Vivo Fiber Photometry Recording

After two weeks of postsurgical recovery and adaptation, recordings were obtained with a THINKERTECH fiber‐photometry system. A two‐channel configuration enabled excitation at 480 nm to capture calcium/green signals and at 405 nm for a reference channel; the reference trace was used to control for motion noise. Analog voltage signals were digitized at 100 Hz using a fiber photometry DAQ digitizer, and FiberPhotometry_dual_color_v2.0.vi (THINKERTECH) handled data logging. The laser output was tuned within 40–70 µW. Use the following methods to analyze the fiber photometric results: In the experiment of Ca^2+^ indicator and H_2_S probe, we calculate the value of fluorescence change (ΔF/F), which is defined as (ΔF/F) = (F‐F0)/(F0‐Fbaseline). F is defined as the real‐time value of fluorescence signal after the optical fiber recording system is connected to the mouse head, and F0 is defined as the average fluorescence signal value after the optical fiber recording system is connected to the mouse head.

### 4.14 Immunofluorescence

Mice were deeply anesthetized with pentobarbital sodium (50 mg/kg, i.p.) and transcardially perfused with ice‐cold phosphate‐buffered saline (PBS, 0.1 M, pH 7.4) to flush out the blood. Once the circulation was cleared, 4% paraformaldehyde (PFA) was perfused to achieve tissue fixation. Brains were carefully dissected, post‐fixed in 4% PFA at 4°C overnight, and then dehydrated in a 30% sucrose solution at 4°C overnight. The brains were subsequently embedded in optimal cutting temperature (OCT) compound (Sakura) and stored at −20°C. Coronal sections (40 µm thick) containing the anterior cingulate cortex (ACC) and optical fiber tracks were prepared using a freezing microtome (MNT, SLEE, Mainz).

Tissue sections were permeabilized in PBS containing 0.3% Triton X‐100 (BS084, Biosharp) for 30 min and then blocked with 5% bovine serum albumin in PBS for 1 h at room temperature. Slices were incubated overnight at 4°C with primary antibodies, including rabbit anti‐glutamate (1:500, G6642, Sigma–Aldrich) and mouse anti‐c‐Fos (1:500, sc‐166940, Santa Cruz Biotechnology). After thorough PBS washes, sections were incubated with species‐specific fluorophore‐conjugated secondary antibodies at 37°C for 1 h in the dark: Alexa Fluor 568‐conjugated goat anti‐rabbit IgG (1:500, ab175471, Abcam) and Alexa Fluor 488‐conjugated goat anti‐mouse IgG (1:500, ab150113, Abcam). Nuclear staining was performed using DAPI (BL105A, Biosharp) for 10 min at room temperature in the dark.Following a final wash, sections were mounted on microscope slides using anti‐fade mounting medium (BL701A, Biosharp) and stored at −20°C until imaging. Fluorescence images were acquired using a confocal microscope (LSM880, Zeiss) and the TissueFAXS imaging system (TissueGnostics).

### 4.15 Validating Bloodstream Delivery of H_2_S From Ectopic Lesions to the ACC

After 21 days of endometriosis model induction, mice were administered an intraperitoneal injection of either 100 µL PBS or 100 µL ACS14@BSA nanoparticles. Eight hours later, 250 µL of 1 mM Washington State Probe‐1 (H_2_S specific fluorescent probe), a reaction‐based turn‐on fluorescent probe containing a reactive disulfide moiety that selectively and rapidly reacts with H_2_S to form benzodithiolone and a fluorescent product (Ex = 465 nm, Em = 515 nm) (MX5301, Shanghai Maokang Biotechnology Co., Ltd.) was injected intraperitoneally. Following this, ectopic lesions, eutopic endometrium, and major organs (heart, liver, spleen, lungs, and kidneys) were harvested for fluorescence imaging using an IVIS system. Subsequently, the lesions were embedded in OCT compound and stored at −20°C. The lesions were cryosectioned at a thickness of 10 µm using a cryostat. After washing with PBS, the sections were stained with DAPI for 10 min in the dark. Following the final wash, the sections were mounted on microscope slides and stored at −20°C until imaging. Fluorescence images were acquired using a confocal microscope and a TissueFAXS imaging system.

To monitor H_2_S delivery to the anterior cingulate cortex (ACC), a guide cannula (RWD Life Science) was implanted above the ACC, with an optical fiber inserted obliquely beneath the cannula for fluorescence signal detection. On day 21 post‐modeling, mice were again injected intraperitoneally with either PBS or ACS14@BSA nanoparticles. After an 8 h period, 200 nL of H_2_S probe (1 mM, WSP‐1) was microinjected via a single‐injector cannula, which extended 0.5 mm beyond the guide cannula tip. Fluorescence signals from H_2_S were recorded in real‐time using a fiber photometry system (THINKERTECH).

### 4.16 Behavioral Procedures

One hour before testing, the mice were acclimated to the experimental environment. The Elevated Plus Maze (EPM) apparatus, consisting of two open arms and two enclosed arms with a central platform, was used to assess anxiety‐like behavior. Mice were placed in the center of the maze, facing one of the open arms and allowed to explore freely for 5 min. Afterward, each arm of the maze was cleaned with a 70% ethanol solution. The number of entries into the open arms and the time spent there were analyzed using Any‐Maze software (Stoelting Co., Wood Dale, IL). Following the EPM, the Open Field Test (OFT) was performed to assess locomotor activity and anxiety‐like behavior in a 50 cm × 50 cm × 40 cm open field apparatus. Mice were placed in the central area and allowed to explore for 5 min, with the apparatus cleaned afterward. Behavioral parameters, including total distance traveled, frequency of entries into the central area, and time spent there, were recorded using Any‐Maze software.

### 4.17 Enzyme‐Linked Immunosorbent Assay (ELISA)

After the completion of the behavioral experiment, the mice were anesthetized, and blood was collected via the medial canthus vein. The blood was then transferred into 1.5 mL plastic tubes and centrifuged at 12 000 rpm for 20 min. The serum was carefully separated and stored at −80°C until further analysis. After blood collection, the mice were euthanized. Corticosterone levels in the serum were quantified using an ELISA kit (CSB‐E07969m, CUSABIO) according to the manufacturer's instructions. Absorbance was measured using a microplate reader (Infinite M200 Pro, Tecan) at the appropriate wavelength.

### 4.18 Western Blotting

After the treatments, the ACC tissue was collected and lysed in sample buffer, followed by heating at 95°C for 10 min. Protein samples were separated using sodium dodecyl sulfate‐polyacrylamide gel electrophoresis (SDS‐PAGE) and then transferred to nitrocellulose membranes. The membranes were incubated overnight at 4°C with a rabbit monoclonal anti‐GLT‐1 antibody (1:1000, ab205248, Abcam), followed by washing. Next, the membranes were exposed to a horseradish peroxidase‐conjugated secondary antibody (1:10000, W4011, Promega) for 1 h at 37°C. After additional washing, chemiluminescent detection was performed using an ECL kit (BL520A, Biosharp), and protein bands were visualized with a chemiluminescence imaging system (Amersham Imager 600, GE Healthcare).

### 4.19 High‐Performance Liquid Chromatography (HPLC)

Glutamate level in the ACC of mice were quantified using a Sciex Triple Quad 4500 HPLC system equipped with a Thermo HYPERSIL GOLD C18 column (4.6 × 250 mm, 5 µm). ACC tissues were rapidly collected and homogenized in 0.4 mol·L^−^^1^ perchloric acid (1:10, w/v) on ice. Homogenates were centrifuged at 10 000 rpm for 15 min at 4°C, and supernatants were neutralized with 4% sodium bicarbonate, filtered through a 0.45 µm membrane, and stored at −80°C until analysis. Prior to injection, samples were derivatized with o‐phthalaldehyde (OPA) and 2‐mercaptoethanol in borate buffer (pH 9.18) at 20°C for 3 min. Chromatographic separation was performed using an isocratic elution system with sodium acetate buffer (pH 6.8), methanol, and tetrahydrofuran (A: 82:17:1, B: 22:77:1) at a flow rate of 1.0 mL/min. Fluorescence detection was carried out with an excitation wavelength of 338 nm and an emission wavelength of 425 nm.

### 4.20 Statistical Analysis

Data are presented as mean ± SEM. Statistical significance was assessed using one‐way analysis of variance (ANOVA) followed by Tukey's post hoc test for comparisons among three or more groups, and two‐tailed Student's *t*‐test for comparisons between two groups. P < 0.05 was considered statistically significant. All analyses were performed using GraphPad Prism 9.

## Author Contributions

**Conceptualization**: S.Z., J.Z, and Y.Z. **Methodology**: S.Z., Y.Z., J.Z., Y.M., J. Q., M.Z., R.D., and R.W. **Investigation**: M.Z., R.D., R.W., Y.X., Y.W., P.W., J.L., and P.L. **Visualization**: M.Z., R.D. Supervision: S.Z., J.Z, and Y.Z. **Writing – original draft**: M.Z., R.D., and R.W. Writing – review and editing: S.Z., J.Z., and Y.Z.

## Conflicts of Interest

The authors declare no conflicts of interest.

## Supporting information




**Supporting File**: advs74156‐sup‐0001‐SuppMat.docx.

## Data Availability

The data that support the findings of this study are available from the corresponding author upon reasonable request.

## References

[1] Y. Wang , K. Nicholes , and I. M. Shih , “The Origin and Pathogenesis of Endometriosis,” Annual Review of Pathology: Mechanisms of Disease 15 (2020): 71–95, 10.1146/annurev-pathmechdis-012419-032654. PMC7980953 31479615

[2] K. T. Zondervan , C. M. Becker , K. Koga , S. A. Missmer , R. N. Taylor , and V. P. Endometriosis , “Endometriosis,” Nature Reviews Disease Primers 4, no. 1 (2018): 9, 10.1038/s41572-018-0008-5. 30026507

[3] S. W. Guo , “Recurrence of Endometriosis and Its Control,” Human Reproduction Update 15, no. 4 (2009): 441–461, 10.1093/humupd/dmp007. 19279046

[4] A. S. Moses , A. A. Demessie , O. Taratula , et al., “Nanomedicines for Endometriosis: Lessons Learned From Cancer Research,” Small 17, no. 7 (2021): 2004975, 10.1002/smll.202004975. PMC7928207 33491876

[5] C. Volpini , N. Bloise , M. Dominoni , et al., “The Nano‐revolution in the Diagnosis and Treatment of Endometriosis,” Nanoscale 15, no. 43 (2023): 17313–17325, 10.1039/D3NR03527A. 37874212

[6] S. Zhu , J. Zhang , N. Xue , et al., “Highly Specific Neutrophil‐mediated Delivery of Albumin Nanoparticles to Ectopic Lesion for Endometriosis Therapy,” Journal of Nanobiotechnology 21, no. 1 (2023): 81, 10.1186/s12951-023-01831-4. 36890521 PMC9996962

[7] M. Zhang , Y. Ye , Z. Chen , et al., “Targeting Delivery of mifepristone to Endometrial Dysfunctional Macrophages for Endometriosis Therapy,” Acta Biomaterialia 189 (2024): 505–518, 10.1016/j.actbio.2024.09.037. 39341437

[8] G. Murphy , D. J. Brayden , D. L. Cheung , A. Liew , M. Fitzgerald , and A. Pandit , “Albumin‐based Delivery Systems: Recent Advances, Challenges, and Opportunities,” Journal of Controlled Release 380 (2025): 375–395, 10.1016/j.jconrel.2025.01.035. 39842723

[9] A. J. Wagner , V. Ravi , R. F. Riedel , et al., “Phase II Trial of Nab‐Sirolimus in Patients with Advanced Malignant Perivascular Epithelioid Cell Tumors (AMPECT): Long‐Term Efficacy and Safety Update,” Journal of Clinical Oncology 42, no. 13 (2024): 1472–1476, 10.1200/JCO.23.02266. 38427923 PMC11095855

[10] D. Koller , G. A. Pathak , F. R. Wendt , et al., “Epidemiologic and Genetic Associations of Endometriosis with Depression, Anxiety, and Eating Disorders,” JAMA Network Open 6, no. 1 (2023): 2251214, 10.1001/jamanetworkopen.2022.51214. PMC9856929 36652249

[11] A. S. Laganà , V. L. La Rosa , A. M. C. Rapisarda , et al., “Anxiety and Depression in Patients With Endometriosis: Impact and Management Challenges,” International Journal of Women's Health 9 (2017): 323–330, 10.2147/IJWH.S119729. PMC5440042 28553145

[12] P. S. Thiel , O. Bougie , J. Pudwell , J. Shellenberger , M. P. Velez , and A. Murji , “Endometriosis and Mental Health: A Population‐based Cohort Study,” American Journal of Obstetrics and Gynecology 230, no. 6 (2024): p649.e1–649.e19, 10.1016/j.ajog.2024.01.023. 38307469

[13] E. Rasp , L. Saavalainen , A. But , et al., “Psychiatric Disorders and Mortality due to External Causes Following Diagnosis of Endometriosis at a Young Age: A Longitudinal Register‐based Cohort Study in Finland,” American Journal of Obstetrics and Gynecology 230, no. 6 (2024): 651.e1–651.e17, 10.1016/j.ajog.2024.02.011. 38365101

[14] C. Locher , H. Koechlin , S. R. Zion , et al., “Efficacy and Safety of Selective Serotonin Reuptake Inhibitors, Serotonin‐Norepinephrine Reuptake Inhibitors, and Placebo for Common Psychiatric Disorders Among Children and Adolescents,” JAMA Psychiatry 74, no. 10 (2017): 1011–1020, 10.1001/jamapsychiatry.2017.2432. 28854296 PMC5667359

[15] A. Slee , I. Nazareth , P. Bondaronek , Y. Liu , Z. Cheng , and N. Freemantle , “Pharmacological Treatments for Generalised Anxiety Disorder: A Systematic Review and Network Meta‐analysis,” The Lancet 393, no. 10173 (2019): 768–777, 10.1016/S0140-6736(18)31793-8. 30712879

[16] I. V. Hofe , B. H. Stricker , M. W. Vernooij , M. K. Ikram , M. A. Ikram , and F. J. Wolters , “Benzodiazepine Use in Relation to Long‐Term Dementia Risk and Imaging Markers of Neurodegeneration: A Population‐based Study,” BMC Medicine 22, no. 1 (2024): 266, 10.1186/s12916-024-03437-5. 38951846 PMC11218055

[17] H. Ding , J. Chang , F. He , S. Gai , and P. Yang , “Hydrogen Sulfide: An Emerging Precision Strategy for Gas Therapy,” Advanced Healthcare Materials 11, no. 4 (2022): 2101984, 10.1002/adhm.202101984. 34788499

[18] C. Szabo , “Gasotransmitters in Cancer: From Pathophysiology to Experimental Therapy,” Nature Reviews Drug Discovery 15, no. 3 (2016): 185–203, 10.1038/nrd.2015.1. 26678620 PMC5319818

[19] J. L. Wallace and R. Wang , “Hydrogen Sulfide‐Based Therapeutics: Exploiting a Unique but Ubiquitous Gasotransmitter,” Nature Reviews Drug Discovery 14, no. 5 (2015): 329–345, 10.1038/nrd4433. 25849904

[20] T. Pandey , R. S. Kaundal , and V. Pandey , “Mechanisms of Hydrogen Sulfide‐Mediated Neuroprotection: Current Understanding and Future Directions,” Neuroscience and Behavioral Physiology 54, no. 8 (2024): 1105–1120, 10.1007/s11055-024-01690-y.

[21] J. Zhang , P. Wang , M. Zhou , et al., “Gasotransmitter‐Nanodonor for Spatial Regulation of Anxiety‐Like Behavior and Bone Metastasis,” Advanced Materials 37, no. 15 (2025): 2416481, 10.1002/adma.202416481. 40042445

[22] A. Sparatore , E. Perrino , V. Tazzari , et al., “Pharmacological profile of a novel H_2_S‐Releasing aspirin,” Free Radical Biology and Medicine 46, no. 5 (2009): 586–592, 10.1016/j.freeradbiomed.2008.11.013. 19100325

[23] H. Zhang , L.‐Z. Hao , J.‐A. Pan , et al., “Microfluidic Fabrication of Inhalable Large Porous Microspheres Loaded With H_2_S‐Releasing Aspirin Derivative for Pulmonary Arterial Hypertension Therapy,” Journal of Controlled Release 329 (2021): 286–298, 10.1016/j.jconrel.2020.11.060. 33279605

[24] B. Lu , X. Han , A. Zhao , et al., “Intelligent H_2_S Release Coating for Regulating Vascular Remodeling,” Bioactive Materials 6, no. 4 (2021): 1040–1050. 33102945 10.1016/j.bioactmat.2020.09.023 PMC7567040

[25] S. S. Wang , Y. H. Chen , N. Chen , et al., “Hydrogen Sulfide Promotes Autophagy of Hepatocellular Carcinoma Cells Through the PI3K/Akt/mTOR Signaling Pathway,” Cell Death and Disease 8, no. 3 (2017): 2688, 10.1038/cddis.2017.18. PMC5386547 28333142

[26] Q. Dong , B. Yang , J.‐G. Han , et al., “A Novel Hydrogen Sulfide‐Releasing Donor, HA‐ADT, Suppresses the Growth of Human Breast Cancer Cells Through Inhibiting the PI3K/AKT/mTOR and Ras/Raf/MEK/ERK Signaling Pathways,” Cancer Letters 455 (2019): 60–72, 10.1016/j.canlet.2019.04.031. 31042588

[27] M. J. Yin , Y. Yamamoto , and R. B. Gaynor , “The Anti‐Inflammatory Agents Aspirin and Salicylate Inhibit the Activity of IκB Kinase‐β,” Nature 396, no. 6706 (1998): 77–80, 10.1038/23948. 9817203

[28] L. József , C. Zouki , N. A. Petasis , C. N. Serhan , and J. G. Filep , “Lipoxin A4 and Aspirin‐Triggered 15‐Epi‐Lipoxin A4 Inhibit Peroxynitrite Formation, NF‐Kappa B and AP‐1 Activation, and IL‐8 Gene Expression in human Leukocytes,” Proceedings of the National Academy of Sciences (PNAS) 99, no. 20 (2002): 13266–13271. 10.1073/pnas.202296999 PMC130622 12235371

[29] H. Yu , L. Lin , Z. Zhang , H. Zhang , and H. Hu , “Targeting NF‐κB Pathway for the Therapy of Diseases: Mechanism and Clinical Study,” Signal Transduction and Targeted Therapy 5, no. 1 (2020): 209, 10.1038/s41392-020-00312-6. 32958760 PMC7506548

[30] M. W. Laschke and M. D. Menger , “Basic Mechanisms of Vascularization in Endometriosis and Their Clinical Implications,” Human Reproduction Update 24, no. 2 (2018): 207–224, 10.1093/humupd/dmy001. 29377994

[31] J. E. Girling , “Harnessing the Inflammatory Processes in Endometriosis,” Nature Reviews Endocrinology 20, no. 2 (2024): 69–70, 10.1038/s41574-023-00937-x. 38062120

[32] G. Batalle , X. Bai , E. Pouso‐Vazquez , G. Roch , L. Rodriguez , and O. Pol , “The Recovery of Cognitive and Affective Deficiencies Linked With Chronic Osteoarthritis Pain and Implicated Pathways by Slow‐Releasing Hydrogen Sulfide Treatment,” Antioxidants (Basel) 10, no. 10 (2021). 10.3390/antiox10101632 PMC8533578 34679766

[33] E. Habibitabar , H. Moridi , H. Shateri , et al., “Chronic NaHS Treatment Improves Spatial and Passive Avoidance Learning and Memory and Anxiety‐Like Behavior and Decreases Oxidative Stress in Rats Fed With a High‐Fat Diet,” Brain Research Bulletin 164 (2020): 380–391, 10.1016/j.brainresbull.2020.09.007. 32942011

[34] A. Drapala , D. Koszelewski , L. Tomasova , et al., “Parenteral Na_2_S, a Fast‐Releasing H_2_S Donor, but not GYY4137, A Slow‐Releasing H_2_S donor, Lowers Blood Pressure in Rats,” Acta Biochimica Polonica 64, no. 3 (2017): 561–566, 10.18388/abp.2017_1569. 28753683

[35] Y. Zhao , H. Wang , and M. Xian , “Cysteine‐Activated Hydrogen Sulfide (H_2_S) Donors,” Journal of the American Chemical Society 133, no. 1 (2011): 15–17, 10.1021/ja1085723. 21142018 PMC3073703

[36] J. Maddern , L. Grundy , A. Harrington , G. Schober , J. Castro , and S. M. Brierley , “A Syngeneic Inoculation Mouse Model of Endometriosis That Develops Multiple Comorbid Visceral and Cutaneous Pain Like Behaviours,” Pain 163, no. 8 (2022): 1622–1635, 10.1097/j.pain.0000000000002552. 35050959

[37] M. Zhuo , “Neural Mechanisms Underlying Anxiety–Chronic Pain Interactions,” Trends in Neurosciences 39, no. 3 (2016): 136–145, 10.1016/j.tins.2016.01.006. 26878750

[38] S. H. Journee , V. P. Mathis , C. Fillinger , P. Veinante , and I. Yalcin , “Janus Effect of the Anterior Cingulate Cortex: Pain and Emotion,” Neuroscience and Biobehavioral Reviews 153 (2023): 105362, 10.1016/j.neubiorev.2023.105362. 37595650

[39] Z. Song , Y. Sun , P. Liu , et al., “Terahertz Wave Alleviates Comorbidity Anxiety in Pain by Reducing the Binding Capacity of Nanostructured Glutamate Molecules to GluA_2_ ,” Research 7 (2024): 0535. 39664293 10.34133/research.0535 PMC11633831

[40] W. Gao , D.‐D. Long , T.‐T. Pan , et al., “Dexmedetomidine Alleviates Anxiety‐Like Behavior in Mice Following Peripheral Nerve Injury by Reducing Thehyperactivity of Glutamatergic Neurons in the Anterior Cingulate Cortex,” Biochemical Pharmacology 206 (2022): 115293, 10.1016/j.bcp.2022.115293. 36241093

[41] X. Zhang , H. Li , S. Huang , et al., “Endocytic Programming via Porous Silicon Nanoparticles Enhances TLR4 Nanoagonist Potency for Macrophage‐Mediated Immunotherapy,” Advanced Functional Materials 36 (2025): 05459.

[42] A. Thakur , J. Patwa , S. Pant , A. Sharma , and S. J. S. Flora , “Interaction Study of Monoisoamyl Dimercaptosuccinic Acid with Bovine Serum Albumin Using Biophysical and Molecular Docking Approaches,” Scientific Reports 11, no. 1 (2021): 4068, 10.1038/s41598-021-83534-0. 33603022 PMC7892868

[43] H. Yang , S. Yang , J. Kong , A. Dong , and S. Yu , “Obtaining Information About Protein Secondary Structures in Aqueous Solution Using Fourier Transform IR Spectroscopy,” Nature Protocols 10, no. 3 (2015): 382–396, 10.1038/nprot.2015.024. 25654756

[44] C. Rao and R. Venkataraghavan , “The C═S stretching frequency and the “‐NC═S bands” in the infrared,” Spectrochimica Acta 18, no. 4 (1962): 541–547.

[45] Z. Yang , N. Zhang , T. Ma , L. Liu , L. Zhao , and H. Xie , “Engineered Bovine Serum Albumin‐Based Nanoparticles With pH‐Sensitivity for Doxorubicin Delivery and Controlled Release,” Drug Delivery 27, no. 1 (2020): 1156–1164, 10.1080/10717544.2020.1797243. 32755291 PMC7470134

[46] C. Wang , T. Zhao , Y. Li , G. Huang , M. A. White , and J. Gao , “Investigation of Endosome and Lysosome Biology by Ultra pH‐Sensitive Nanoprobes,” Advanced Drug Delivery Reviews 113 (2017): 87–96, 10.1016/j.addr.2016.08.014. 27612550 PMC5339051

[47] C. Xie , D. Cen , Z. Ren , et al., “FeS@BSA Nanoclusters to Enable H_2_S‐Amplified ROS‐Based Therapy With MRI Guidance,” Advanced Science 7, no. 7 (2020): 1903512, 10.1002/advs.201903512. 32274323 PMC7141047

[48] E. Somigliana , P. Vigano , G. Rossi , S. Carinelli , M. Vignali , and P. Panina‐Bordignon , “Endometrial Ability to Implant in Ectopic Sites Can be Prevented by Interleukin‐12 in a Murine Model of Endometriosis,” Human Reproduction 14, no. 12 (1999): 2944–2950, 10.1093/humrep/14.12.2944. 10601076

[49] M. Zhou , Y. Wang , C. Li , et al., “Neutrophil Hitchhiking Liposomal Drugs for Starvation Therapy in Endometriosis,” Theranostics 15, no. 10 (2025): 4848–4860, 10.7150/thno.107758. 40225582 PMC11984412

[50] G. Cirino , C. Szabo , and A. Papapetropoulos , “Physiological Roles of Hydrogen Sulfide in Mammalian Cells, Tissues, and Organs,” Physiological Reviews 103, no. 1 (2023): 31–276, 10.1152/physrev.00028.2021. 35435014

[51] A. Pałasz , I. C. Menezes , and J. J. Worthington , “The Role of Brain Gaseous Neurotransmitters in Anxiety,” Pharmacological Reports 73, no. 2 (2021): 357–371. 33713315 10.1007/s43440-021-00242-2 PMC7994231

[52] Y. Qiu , M. Fan , Y. Wang , et al., “Sulfate‐Reducing Bacteria Loaded in Hydrogel as a Long‐Lasting H_2_S Factory for Tumor Therapy,” Journal of Controlled Release 360 (2023): 647–659, 10.1016/j.jconrel.2023.06.037. 37406817

[53] Z. Su , L. Kong , Y. Dai , et al., “Bioresponsive Nano‐Antibacterials for H_2_S‐Sensitized Hyperthermia and Immunomodulation Against Refractory Implant–Related Infections,” Science Advances 8, no. 14 (2022): abn1701, 10.1126/sciadv.abn1701. PMC8993125 35394829

[54] P. Houeto , P. Houze , and F. J. Baud , “Comparative Study of the Tissue Distribution of Equimolar Repeated Doses of Hydroxocobalamin and Cobalt Chloride in the Rats,” Annales De Biologie Clinique 76, no. 2 (2018): 179–184. 29623888 10.1684/abc.2017.1318

[55] D. Zhang , B. Lee , A. Nutter , et al., “Protection From Cyanide‐Induced Brain Injury by the Nrf2 Transcriptional Activator Carnosic Acid,” Journal of Neurochemistry 133, no. 6 (2015): 898–908, 10.1111/jnc.13074. 25692407 PMC4465065

[56] E. H. Simpson , T. Akam , T. Patriarchi , et al., “Lights, Fiber, Action! A Primer on In Vivo Fiber Photometry,” Neuron 112, no. 5 (2024): 718–739, 10.1016/j.neuron.2023.11.016. 38103545 PMC10939905

[57] G. Cui , S. B. Jun , X. Jin , et al., “Deep Brain Optical Measurements of Cell Type–Specific Neural Activity in Behaving Mice,” Nature Protocols 9, no. 6 (2014): 1213–1228, 10.1038/nprot.2014.080. 24784819 PMC4100551

[58] L. Cong , S. Ding , Y. Guo , et al., “Acupuncture Alleviates CSDS‐Induced Depressive‐Like Behaviors by Modulating Synaptic Plasticity in vCA1,” Theranostics 15, no. 10 (2025): 4808–4822, 10.7150/thno.106751. 40225589 PMC11984413

[59] M. Nedergaard , T. Takano , and A. J. Hansen , “Beyond the Role of Glutamate as a Neurotransmitter,” Nature Reviews Neuroscience 3, no. 9 (2002): 748–755, 10.1038/nrn916. 12209123

[60] A. Reiner and J. Levitz , “Glutamatergic Signaling in the Central Nervous System: Ionotropic and Metabotropic Receptors in Concert,” Neuron 98, no. 6 (2018): 1080–1098, 10.1016/j.neuron.2018.05.018. 29953871 PMC6484838

[61] E. E. Benarroch , “Glutamate Transporters,” Neurology 74, no. 3 (2010): 259–264, 10.1212/WNL.0b013e3181cc89e3. 20083803

[62] R. J. Vandenberg and R. M. Ryan , “Mechanisms of Glutamate Transport,” Physiological Reviews 93, no. 4 (2013): 1621–1657, 10.1152/physrev.00007.2013. 24137018

[63] M. Lu , L. F. Hu , G. Hu , and J. S. Bian , “Hydrogen Sulfide Protects Astrocytes Against H_2_O_2_‐Induced Neural Injury via Enhancing Glutamate Uptake,” Free Radical Biology and Medicine 45, no. 12 (2008): 1705–1713, 10.1016/j.freeradbiomed.2008.09.014. 18848879

[64] J. F. Wang , Y. Li , and J. N. Song , “Role of Hydrogen Sulfide in Secondary Neuronal Injury,” Neurochemistry International 64 (2014): 37–47, 10.1016/j.neuint.2013.11.002. 24239876

[65] H. Oster , E. Challet , V. Ott , et al., “The Functional and Clinical Significance of the 24‐Hour Rhythm of Circulating Glucocorticoids,” Endocrine Reviews 38, no. 1 (2017): 3–45, 10.1210/er.2015-1080. 27749086 PMC5563520

