# Supplementary material for: H2S‐Releasing Aspirin Nanoparticles Alleviate Endometriosis and Associated Anxiety
Source: Adv Sci (Weinh). 2026 Feb 8;13(20):e20787. doi: 10.1002/advs.202520787 (PMC13067765; doi:10.1002/advs.202520787)
Supplement: Supplementary file 1 — Supporting File: advs74156‐sup‐0001‐SuppMat.docx. [file ADVS-13-e20787-s001.docx]

Supplementary Information

**H₂S-Releasing Aspirin Nanoparticles Alleviate Endometriosis and Associated Anxiety**

*Mengni Zhou^1+^, Renbin Dou^1,5+^, Rong Wu^1+^, Yunyu Xu^1^, Ying Wang^2^, Peng Wang^2^, Jinfu Li^2^, Pengcheng Lu^2^, Yu Mao^2^, Jieying Qian^3,4^, Yunjiao Zhang^3,4^*, Jiqian Zhang^2^*, Shasha Zhu^1^**

1. *Department of Obstetrics and Gynecology, the First Affiliated Hospital of Anhui Medical University, NHC Key Laboratory of Study on Abnormal Gametes and Reproductive Tract (Anhui Medical University), Engineering Research Center of Biopreservation and Artificial Organs, Ministry of Education, No 218 Jixi Road, Hefei 230022, Anhui, China*
2. *Department of Anesthesiology, the First Affiliated Hospital of Anhui Medical University, Key Laboratory of Anesthesiology and Perioperative Medicine of Anhui Higher Education Institutes, Anhui Medical University, No 218 Jixi Road, Hefei 230022, Anhui, China*
3. *School of Medicine, South China University of Technology, Guangzhou 510006, Guangdong, China*
4. *National Engineering Research Center for Tissue Restoration and Reconstruction and Key Laboratory of Biomedical Engineering of Guangdong Province, South China University of Technology, Guangzhou 510006, Guangdong, China*
5. *Lu'an People's Hospital of Anhui Province, Lu'an Hospital of Anhui Medical University**,* *No. 21 Wanxi West Road, Lu'an* *237000, Anhui, China*

*[*+*] These authors contributed equally to this work*

*[*] To whom correspondence should be addressed*

*E-mail: zhangyunjiao@scut.edu.cn; jiqianzh@mail.ustc.edu.cn; zss90328@mail.ustc.edu.cn*

**Table S1**. Entrapment efficiency and drug loading of ACS14@BSA nanoparticles(N=3)

| Formulation | LC (%) | EE (%) |
| --- | --- | --- |
| ACS14@BSA | 10.2 ± 1.3 | 37.8 ± 5.3 |


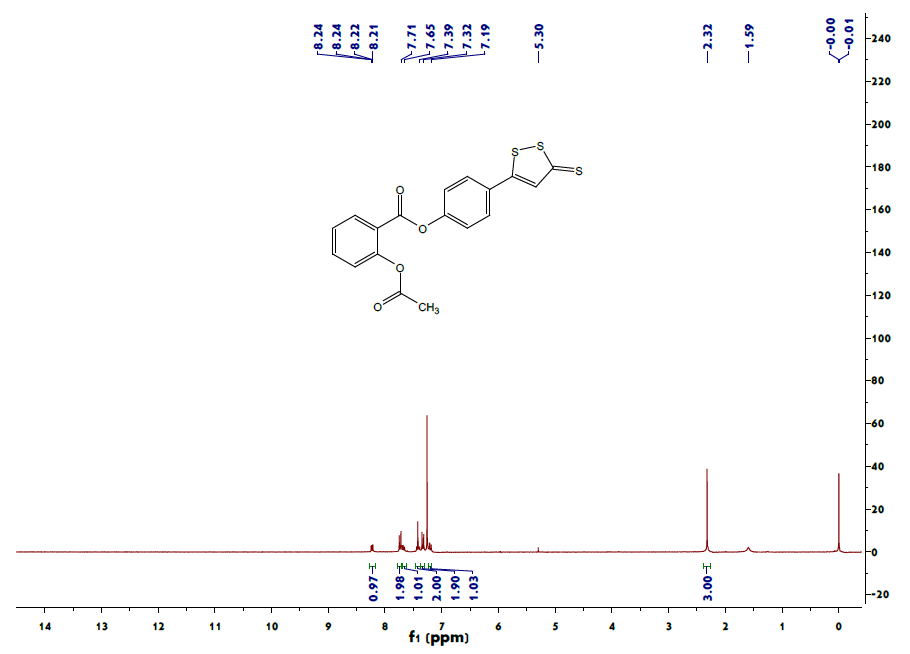


**Figure S1.** ^1^H NMR spectra of ACS14.


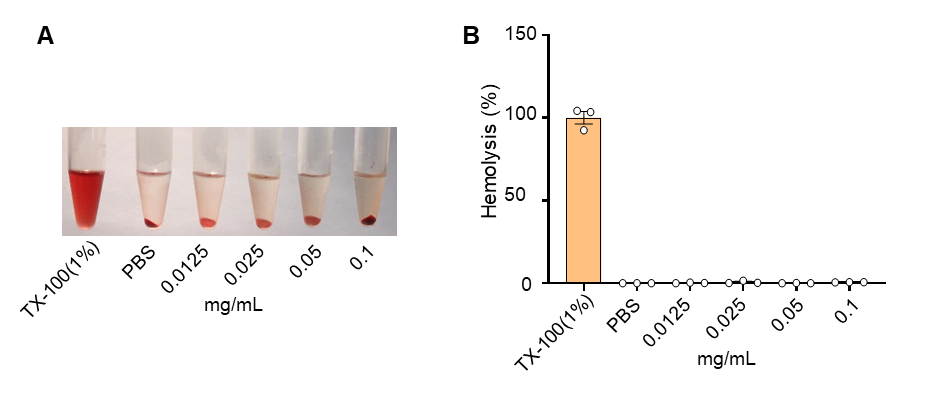


**Figure S2.** (**A**) Representative images of sheep red blood cells and (**B**) hemolysis assay in the presence of 1% Triton X-100 (TX-100), PBS, or various concentrations of ACS14@BSA, respectively. Data are shown as the mean ± SEM. n = 3.


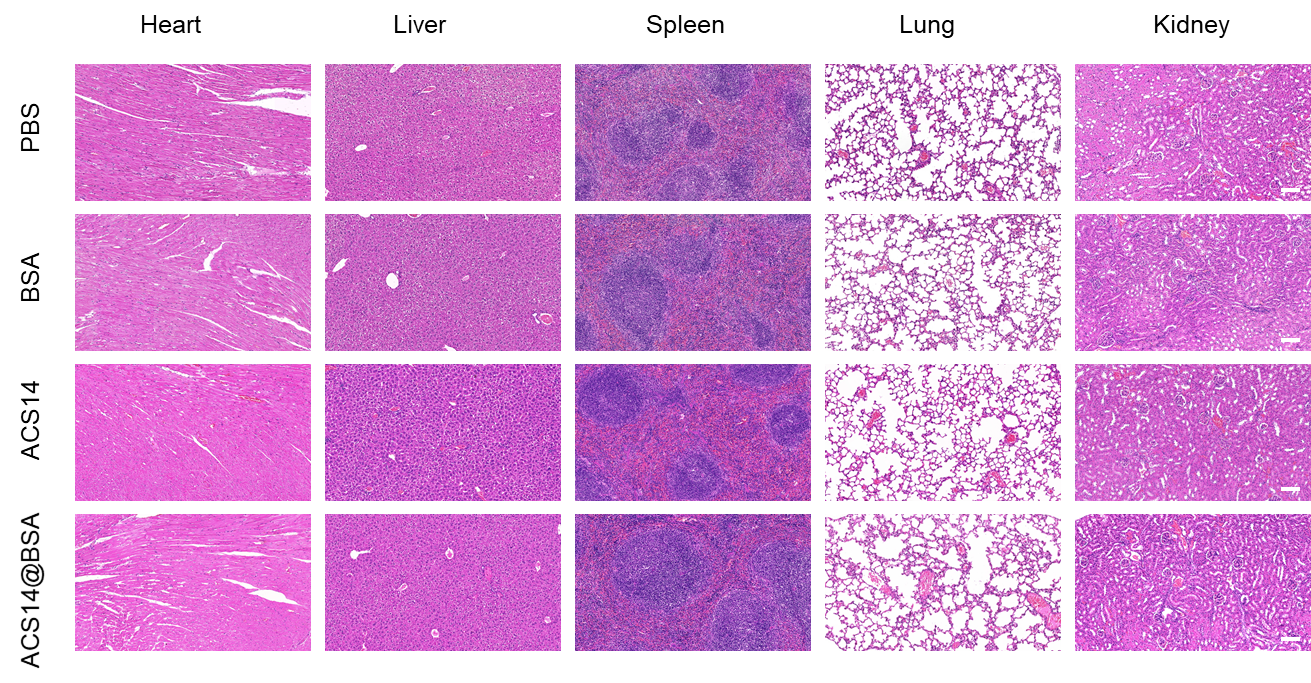


**Figure S3.** Hematoxylin and eosin (H&E) staining of major organs (heart, liver, spleen, lung, and kidney) of mice treated with different formulations. Scale bar: 100 μm.


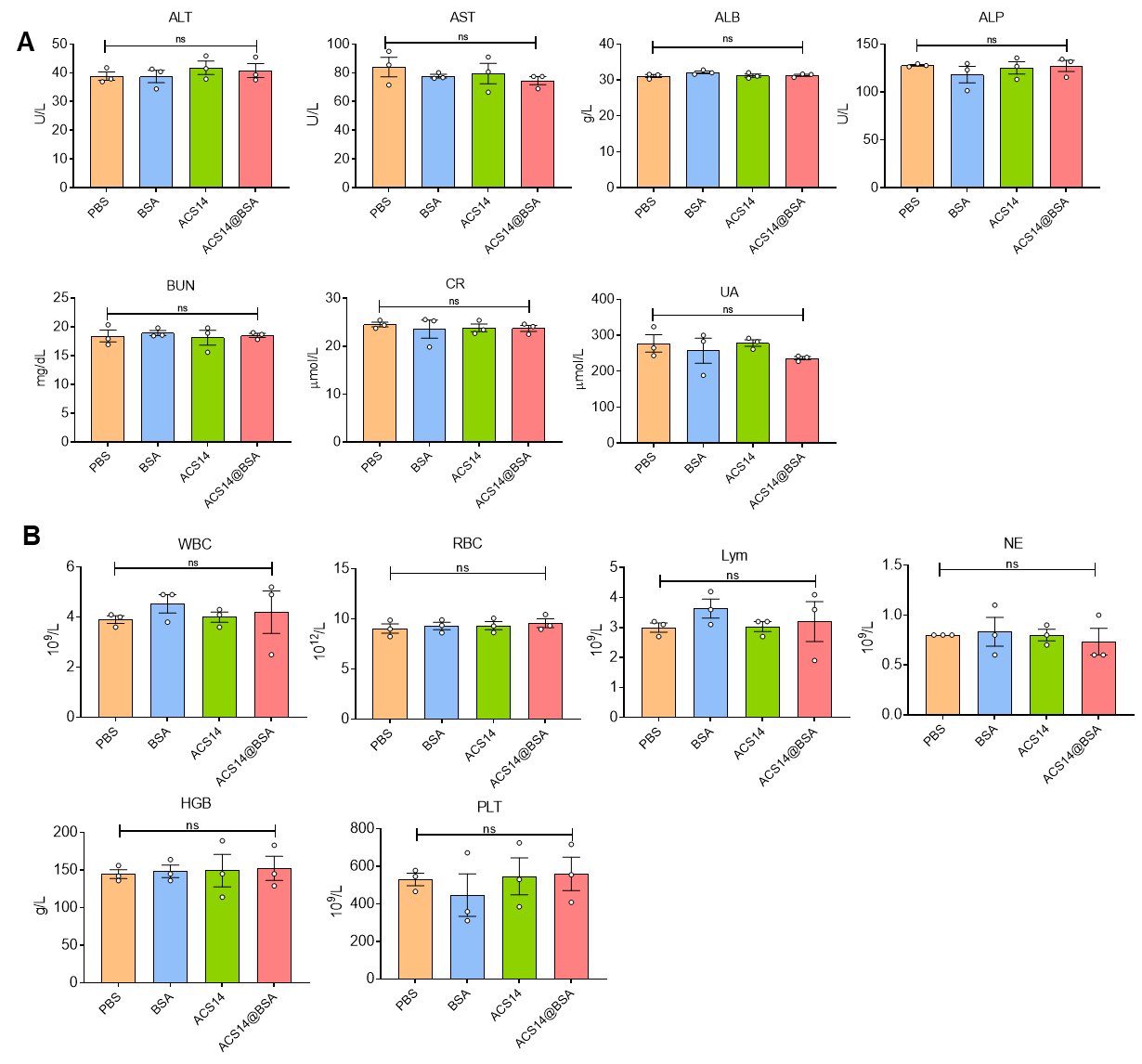


**Figure S4.** (**A**) Blood routine indicators in mouse blood. (**B**) Blood biochemistry indicators in mouse blood. n= 3. Data are presented as mean ± SEM. ns, not significant.


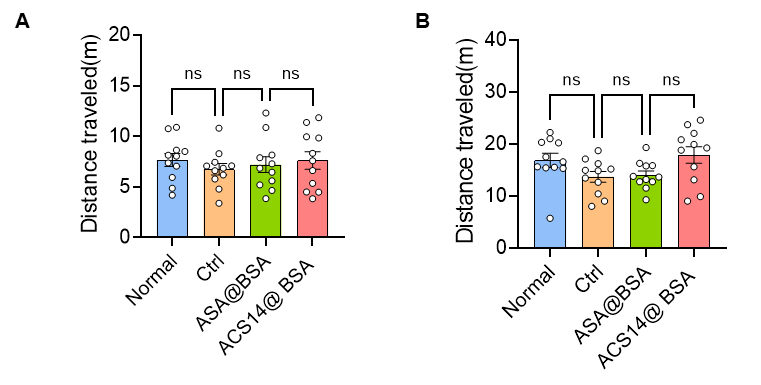


**Figure S5.** The total distance traveled in behavioral test. (**A**) The total distance traveled in EPM. (**B**) The total distance traveled in OFT. n = 11. Data are presented as mean ± SEM. ns, not significant.
